# Inhibiting Ca^2+^ channels in Alzheimer’s disease model mice relaxes pericytes, improves cerebral blood flow and reduces immune cell stalling and hypoxia

**DOI:** 10.1038/s41593-024-01753-w

**Published:** 2024-09-18

**Authors:** Nils Korte, Anna Barkaway, Jack Wells, Felipe Freitas, Huma Sethi, Stephen P. Andrews, John Skidmore, Beth Stevens, David Attwell

**Affiliations:** 1 Department of Neuroscience, Physiology & Pharmacology, University College London, London, UK; 2 UCL Centre for Advanced Biomedical Imaging, Division of Medicine, University College London, London, UK; 3 Department of Neurosurgery, National Hospital for Neurology and Neurosurgery, London, UK; 4 ALBORADA Drug Discovery Institute, University of Cambridge, Cambridge, UK; 5 Present Address: Department of Neurology, Boston Children’s Hospital, Harvard Medical School, Boston, MA USA; 6 Stanley Center, Broad Institute, Cambridge, MA USA

**Keywords:** Alzheimer's disease, Experimental models of disease, Neuro-vascular interactions

## Abstract

Early in Alzheimer’s disease (AD), pericytes constrict capillaries, increasing their hydraulic resistance and trapping of immune cells and, thus, decreasing cerebral blood flow (CBF). Therapeutic approaches to attenuate pericyte-mediated constriction in AD are lacking. Here, using in vivo two-photon imaging with laser Doppler and speckle flowmetry and magnetic resonance imaging, we show that Ca^2+^ entry via L-type voltage-gated calcium channels (CaVs) controls the contractile tone of pericytes. In AD model mice, we identifed pericytes throughout the capillary bed as key drivers of an immune reactive oxygen species (ROS)-evoked and pericyte intracellular calcium concentration ([Ca^2+^]_i_)-mediated decrease in microvascular flow. Blocking CaVs with nimodipine early in disease progression improved CBF, reduced leukocyte stalling at pericyte somata and attenuated brain hypoxia. Amyloid β (Aβ)-evoked pericyte contraction in human cortical tissue was also greatly reduced by CaV block. Lowering pericyte [Ca^2+^]_i_ early in AD may, thus, offer a therapeutic strategy to enhance brain energy supply and possibly cognitive function in AD.

## Abstract (web summary)

Early in Alzheimer’s disease (AD), brain blood flow is reduced by pericytes constricting capillaries. Korte et al. show that oral nimodipine can reverse this and decrease brain hypoxia. Blocking capillary constriction is a potential add-on therapy in AD.

## Main

Most therapies for Alzheimer’s disease (AD) aim to remove amyloid β (Aβ) plaques or prevent tau hyperphosphorylation, but they have failed to prevent cognitive decline^1,2^. This may be because, when therapies are started, irreversible neuronal damage has already occurred, prompting a search for new therapeutic targets that are useful early in the disease. One such target is cerebral blood flow (CBF). CBF is reduced by approximately 45% in affected brain areas in AD^3^, sufficient to result in loss of attention, myelinated axon disruption, spatial memory deficits and synapse loss^4–6^. CBF decrease is associated with increased capillary transit time heterogeneity, which amplifies tissue hypoxia^7^. A causal influence of blood flow changes on cognitive changes early in AD, before synapses or neurons are lost, is suggested by the CBF reduction starting early^8,9^ in preclinical AD, with a faster onset than the deposition of Aβ or tau^10^, and correlating with cognitive decline^7,11^.

In human AD, this CBF decrease is associated with capillary constriction by contractile pericytes^12^. This may reflect Aβ stimulating production of reactive oxygen species (ROS) that release the vasoconstrictor endothelin-1 (ET-1)^12^. Pericyte-mediated capillary constriction is also seen in the APP^NL–G–F^ mouse model of AD, in which there was no constriction of arterioles or venules, implicating pericytes as causing the CBF decrease^12^. Pericyte contraction is evoked by a rise of intracellular calcium concentration ([Ca^2+^]_i_) or by activating the Rho kinase pathway^13–16^. It is best characterized for the 1st–3rd capillary branch orders (from penetrating arterioles (PAs)), for which pericytes have circumferential processes close to their somata^12^, but even pericytes with less circumferential processes on higher-order capillary branches are now thought to be contractile and to regulate CBF^15,17^.

The contraction of pericytes decreases CBF in three ways. First, the capillary diameter reduction that it produces decreases the cross-sectional area available for flow, which increases resistance via Poiseuille’s law. Second, it leads to an increase in blood viscosity, by promoting interaction of cells in the blood with the vessel walls^18^. Finally, blood cells can get stuck in vessels where the diameter is reduced^14,19^. This is especially important for leukocytes, which are larger and less deformable than red blood cells (RBCs).

Agents reducing pericyte contraction in AD should increase CBF by increasing capillary diameter, decreasing blood viscosity and reducing block of capillaries. They might maintain normal neuronal function for longer and delay irreversible deleterious effects on neurons. In the present study, we examined using the blood–brain barrier (BBB)-permeable voltage-gated calcium channel (CaV) blocker nimodipine for this purpose. Combining measurements of pericyte [Ca^2+^]_i_, capillary diameter, blood flow (from laser speckle, laser Doppler and magnetic resonance imaging (MRI)) and capillary stalling, we show that, in an AD mouse model, nimodipine lowers pericyte [Ca^2+^]_i_, thus relaxing pericytes throughout the capillary bed, dilating capillaries and reducing capillary block by neutrophils and other cells. Consequently, CBF is increased and tissue hypoxia reduced.

## Results

**Extended Data Fig. 1:**
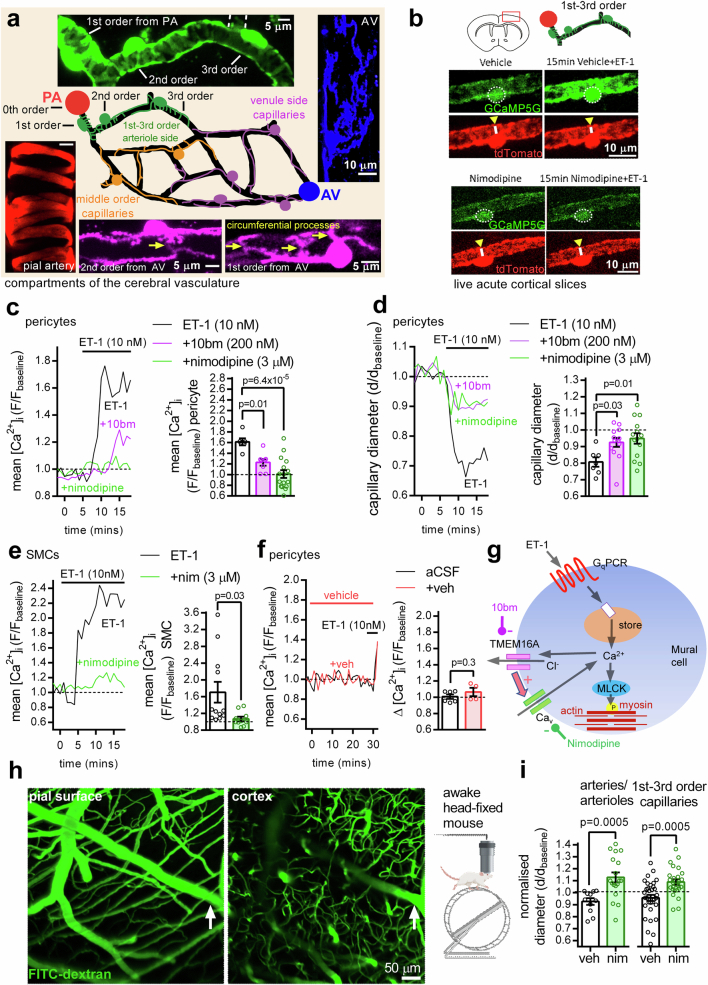
L-type voltage-gated Ca^2+^ channels and Ca^2+^-gated Cl^−^ channels interact to control pericyte contraction. **a** Anatomy of mural cells (defined by NG2-tdTomato label, recolored as below, in NG2-Cre^ERT2^-GCaMP5G-IRES-tdTomato mice) across the cerebral vasculature. SMCs form rings around pial arteries and penetrating arterioles (PAs, red image, red in schematic), pericytes of 1^st^-3^rd^ branching order (green) from PAs wrap circumferential processes around capillaries, pericytes in the middle (orange) and on the venule side (purple, 1^st^-3^rd^ branch order from venule, blue) of the capillary bed show more longitudinal and fewer circumferential processes (Supplementary Video 1). **b** Two-photon microscopy images (maximum intensity projections) of pericytes on 1st-3rd order capillary branches in acute cortical slices from wild-type NG2-Cre^ERT2^-GCaMP5G mice. Endothelin-1 (ET-1) was applied in the absence or presence of nimodipine (CaV blocker) and [Ca^2+^]_i_ in pericyte somata (white dashed circles) and capillary diameter (white line) were measured. **c** Left: time course of ET-1 evoked [Ca^2+^]_i_ rise in pericytes in the absence or presence of nimodipine or 10bm (TMEM16A blocker). Right: nimodipine or 10bm reduce the ET-1 evoked pericyte [Ca^2+^]_i_ rise. P values from one-way ANOVA. **d** Nimodipine and 10bm reduce the ET-1 evoked capillary constriction at pericyte somata. P values from Kruskal^−^Wallis test. **e** Nimodipine reduces ET-1 evoked [Ca^2+^]_i_ rise in SMCs. P value from unpaired t-test. **f** Incubation with vehicle used to dissolve nimodipine does not alter pericyte [Ca^2+^]_i_, but ET-1 applied in last 4 mins of recording raised [Ca^2+^]_i_, confirming pericytes remain responsive to ET-1 during prolonged imaging. P value from unpaired t-test. **g** Proposed mechanism of ET-1 evoked pericyte contraction (see also refs. ^14,79^): ET-1 evoked Ca^2+^ release from stores activates TMEM16A Ca^2+^-gated Cl^−^ channels. The resulting Cl^−^ efflux depolarises the pericyte, which promotes Ca^2+^ entry via CaVs, activating myosin-light chain kinase (MLCK) and pericyte contraction. **h-i** In awake head fixed mature WT mice, imaging (**h**) revealed that nimodipine (220 μg/kg i.v.) dilates arterioles/arteries, and capillaries (**i**). P values from unpaired t-tests. P-values are 2-tailed. Error bars are s.e.m. Source data

The morphology of mural cells (smooth muscle cells (SMCs) and pericytes) at different positions in the capillary bed is shown in Extended Data Fig. 1a. Experiments were initially performed on pericytes of the 1st, 2nd and 3rd capillary branching order from PAs, where 1st order is the first branch off the PA; 2nd order is a branch off the 1st order, etc.

### L-type Ca^2+^ and TMEM16A Cl^−^ channels control pericyte tone

**Extended Data Fig. 2:**
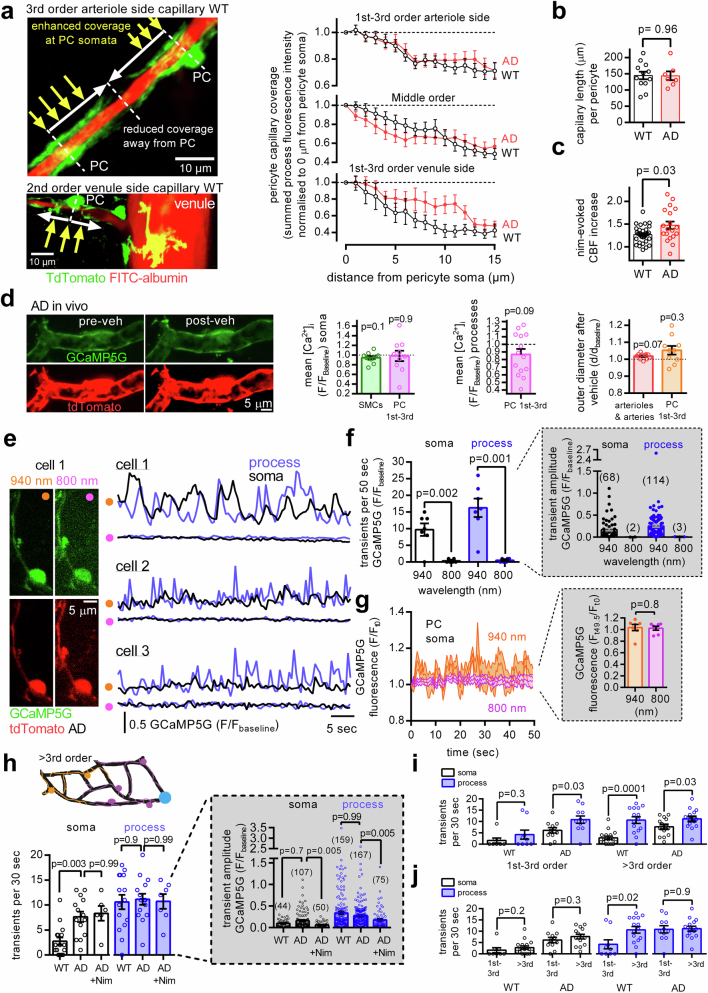
Pericytes show most circumferential process coverage near their somata. **a** Left: arteriole side and venule side pericytes. Right: Pericyte coverage decreases with distance from pericyte soma centre (0 µm), and in AD is enhanced on venule side of capillary bed. Fluorescence intensity of processes at different distances along vessel from soma (averaged across the lumen) was normalised to value at pericyte soma centre (see also ref. ^12^ for counts of circumferential processes versus distance). **b** Length of 3D-traced capillaries (between arteriole and venule) per pericyte did not differ between mature WT (106 pericytes/13337 µm capillary length) and AD mice (84 pericytes/11509 µm capillary length; see also Extended Data Fig. 10f with mature and old mice). **c** Nimodipine evoked CBF increases in mature WT and AD mice regardless of dsRed or NG2-Cre^ERT2^-GCaMP5g co-expression (these data are also plotted in Extended Data Fig. 10a as mature mice). **d** As in Fig. 2d and f, but infusing vehicle for nimodipine. **e-g** GCaMP fluorescence. **e** In vivo GCaMP5g traces of pericytes (cells 1-3, all >3rd branch order) in cortex imaged (2 Hz) at 940 nm (where GCaMP5g signal reflects [Ca^2+^]_i_) or 800 nm (where signal is [Ca^2+^]_i_-independent; Supplementary Video 3). **f** Frequency and amplitude of [Ca^2+^]_i_ transients in somata and processes from images like (e) (one 1st, one 2nd and five >3rd order pericytes). **g** Mean GCaMP fluorescence excited at 940 and 800 nm in pericyte somata of AD mice. **h** Ca^2+^ transient frequency and amplitude in >3rd branch order pericyte processes and somata. **i** Ca^2+^ transient frequency in somata versus processes of 1^st^-3^rd^ or >3^rd^ order pericytes. **j** Ca^2+^ transient frequency in 1^st^-3^rd^ versus >3^rd^ order pericyte somata and processes. P values from unpaired t-tests in panels **b**, **c**, **f**, **g**, **i** (for AD data) and **j** (except for WT soma data), from paired t-tests in **d**, from a Kruskal^−^Wallis test with Dunn’s multiple comparison for **h** except for p-values assessing transient frequency which were from one-way ANOVA, and a Mann-Whitney test for **i** (WT) and **j** (WT soma). P-values are 2-tailed. Error bars are s.e.m. Source data

**Extended Data Fig. 3:**
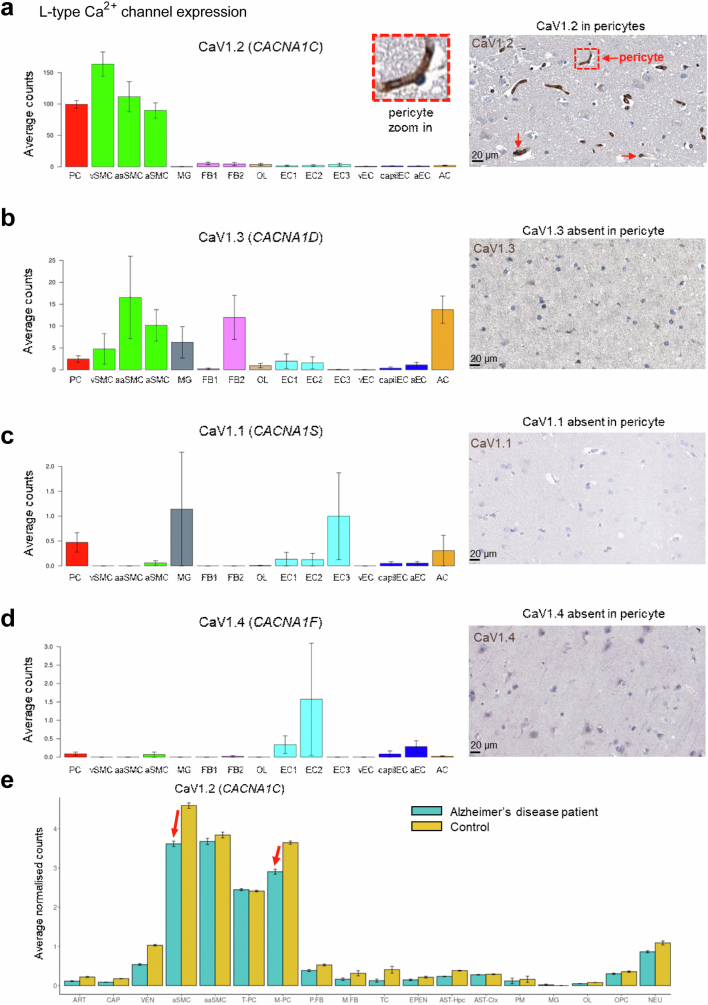
Pericytes and smooth muscle cells express CaV1.2 L-type Ca^2+^ channels. **a-d** Single-cell RNA sequencing in mouse cortex (left bar chart) and histology of human cortex (black labeling on right images), respectively, reveal pericyte (PC) expression of CaV1.2 (**a**) but little or no expression of CaV1.3 (**b**), CaV1.1 (**c**) or CaV1.4 (**d**). Abbreviations: mRNA level (counts), venous smooth muscle cells (vSMCs), arteriolar SMCs (aSMCs), arterial SMCs (aaSMCs), microglia (MG), two fibroblast classes (FB1, FB2), oligodendrocytes (OL), three classes of broadly distributed endothelial cells (EC1-EC3), venous ECs (vEC), capillary ECs (capilEC), arterial ECs (aEC), and astrocytes (AC). Data obtained from refs. ^43,94^ (v23.proteinatlas.org). **e** Single-cell RNA sequencing of the BBB in patients with AD or control individuals (with no cognitive impairment) indicates that CaV1.2 is expressed in pericytes (T-PC, M-PC) and SMCs (aSMC, aaSMC), although with some decrease in AD (red arrows). Other abbreviations: arterial (ART), capillary (CAP), venous (VEN), perivascular fibroblast (P.FB), meningeal fibroblast (M.FB), T cell (TC), Ependymal (EPEN), astrocyte-hippocampus (AST-Hpc), astrocyte-cortex (AST-Ctx), perivascular macrophage (PM), microglia (MG), oligodendrocyte (OL), Oligodendrocyte Precursor Cell (OPC), Neuron (NEU). Data from ref. ^95^. Error bars are s.e.m.

Pericyte contractile tone is controlled by Ca^2+^ via an interaction between L-type CaVs and the Ca^2+^-gated Cl^−^ channel TMEM16A (ref. ^14^). Our strategy for reducing the CBF decrease that occurs early in AD is to prevent a [Ca^2+^]_i_ rise in pericytes. Extended Data Fig. 1b–g shows how blockers of CaVs and TMEM16A can achieve this. Gq-protein-coupled receptor (GqPCR) agonists, such as ET-1 (a driver of Aβ-evoked pericyte contraction in AD^12^), release Ca^2+^ from sarcoplasmic reticulum. This Ca^2+^ may activate actomyosin directly or trigger Cl^−^ exit via TMEM16A channels^14^, leading to membrane depolarization and CaV activation, causing Ca^2+^ influx and contraction. Two-photon imaging of acute cortical slices, from mice with mural cells expressing tdTomato and the calcium indicator GCaMP5g (NG2-Cre^ERT2^-GCaMP5g mice), was performed. ET-1 increased pericyte [Ca^2+^]_i_ and constricted capillaries at pericyte somata (Extended Data Fig. 1b–d) where most circumferential pericyte processes mediating constriction^12^ are present (Extended Data Fig. 2a and Supplementary Video 1). Blocking CaVs with nimodipine, or TMEM16A with 10bm (ref. ^20^), greatly attenuated the [Ca^2+^]_i_ rise and pericyte contraction (Extended Data Fig. 1b–d), and a similar decrease by nimodipine of the ET-1 evoked [Ca^2+^]_i_ rise was detected in SMCs (Extended Data Fig. 1e), consistent with Ca_V_1.2 (Extended Data Fig. 3) and TMEM16A (ref. ^14^) being highly expressed in mural cells. The vehicle used to dissolve nimodipine did not modulate [Ca^2+^]_i_ in pericytes (Extended Data Fig. 1f). Our model, underpinning this paper, of how TMEM16A and CaVs control pericyte tone is shown in Extended Data Fig. 1g.

### CaVs generate myogenic tone in SMCs and pericytes in vivo

**Fig. 1:**
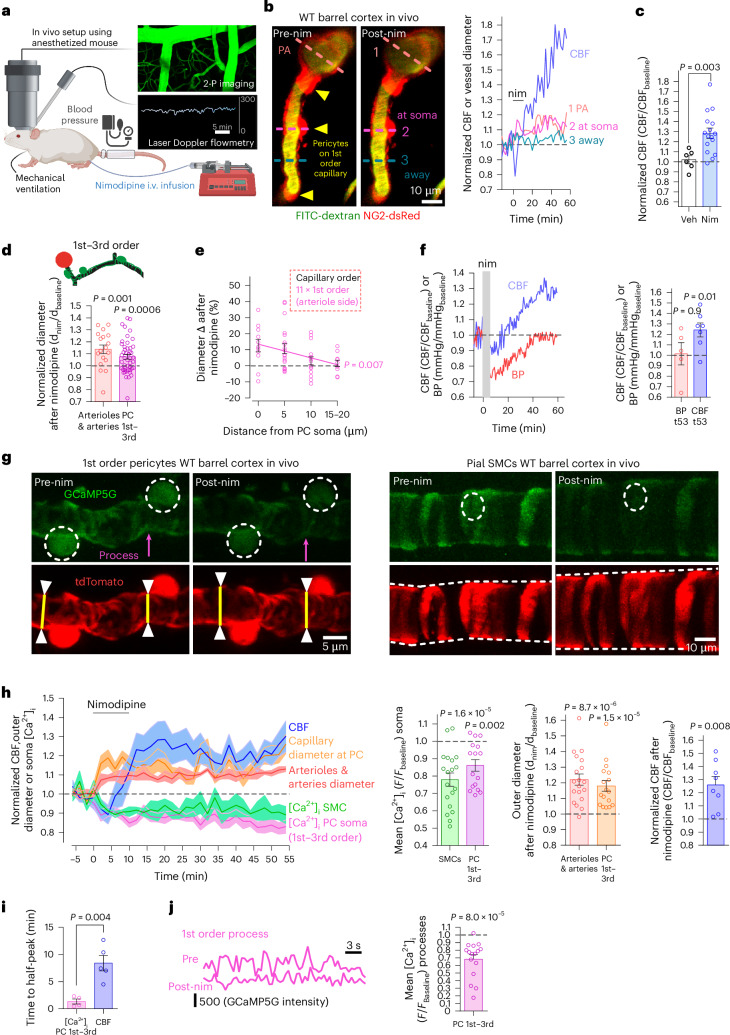
CaVs generate tone in SMCs and 1st–3rd order pericytes in WT mice in vivo. **a**, Barrel cortex of anesthetized intubated mouse: FITC-dextran (green) in blood, laser Doppler for CBF, tail cuff measures BP (created with BioRender). **b**, Left, imaging of FITC-dextran in PA and 1st order capillary before and after i.v. nim in NG2-dsRed mouse (arrows: pericytes). Right, diameters at dashed lines on left, and CBF. **c**, Nim raises CBF compared to vehicle (veh). *P* value: Mann–Whitney test. **d**, Normalized diameter of pial arteries/PAs and capillaries at 1st–3rd order pericytes (0–5 μm from soma center) after nim (schematic from Extended Data Fig. 1a). **e**, Nim-evoked change in 1st order capillary diameter is largest at pericyte somata. *P* value assesses if the line slope is non-zero. **f**, Left, specimen nim-evoked changes in BP and CBF. Right, mean CBF (1–2 points per animal with 1–2 Doppler probes) and BP (one point per animal) after i.v. nim. Shortest recording was 53 min, so this timepoint (t53) was plotted for all traces. **g**, In vivo two-photon images of 1st order capillary pericytes and pial artery SMCs expressing GCaMP5G and tdTomato in NG2-Cre^ERT2^-GCaMP5G mice. Yellow lines, capillary outer diameter; magenta arrow, nim-evoked [Ca^2+^]_i_ drop in process. **h**, Left, normalized (mean ± s.e.m.) change in CBF, diameter of arterioles and capillaries and [Ca^2+^]_i_ in SMCs and pericytes, after nim. Bar graphs: (left) nim-evoked fall in [Ca^2+^]_i_ evoking (middle) vasodilation at 1st–3rd order pericytes and SMCs, facilitating (right) CBF increases in NG2-Cre^ERT2^-GCaMP5G mice (as in NG2-dsRed or WT mice: **c**,**f**). Bars include some data acquired before and after nim without the whole timecourse being defined. **i**, Time to half-peak change of [Ca^2+^]_i_ or CBF for **h**. **j**, Left, nim reduces mean [Ca^2+^]_i_ in the pericyte process (pre-/post-nim traces start ~2,500/2,000 units above zero, respectively). Right, mean [Ca^2+^]_i_ in processes in nim relative to baseline. *P* values are from paired *t*-tests in **d**,**f**,**h**,**j** (for SMC and process [Ca^2+^]_i_, arteriole/artery diameter and CBF), Wilcoxon test in **h** (pericyte [Ca^2+^]_i_ and diameter) and unpaired *t*-test in **i**. *P* values are two-tailed. Error bars: s.e.m. 2-P, two-photon; nim, nimodipine; Veh, vehicle. Source data

Our understanding of pericyte contraction comes mainly from experiments on brain slices. In vivo, intravascular pressure and shear stress release vasoactive messengers from endothelial cells and may cause SMCs and pericytes to constrict vessels. To assess whether CaVs generate contractile tone in vivo, we measured CBF using laser Doppler flowmetry with in vivo two-photon imaging of the cerebral vasculature in urethane-anesthetized NG2-dsRed mice that have pericytes and SMCs labeled with dsRed^21^ (Fig. 1a,b). FITC-dextran (70 kDa, intravenous (i.v.)) was used to visualize blood flow. Femoral vein infusion of the BBB-permeable CaV blocker nimodipine (220 μg kg^−1^ total, over 10 min, 60 μg ml^−1^ solution) evoked a long-lasting increase in CBF of 28% in nine anesthetized mature P120–P158 wild-type (WT) mice (Fig. 1b,c; ‘mature’ is used for mice aged P110–P200). Infusion of vehicle alone did not alter CBF (Fig. 1c), implying that nimodipine, rather than the vehicle or the small increase in blood volume, raises the CBF.

Two-photon imaging of intraluminal FITC-dextran (Fig. 1b) showed that nimodipine-evoked CBF rises coincided with a long-lasting increase in the diameters of pial arteries, penetrating arterioles and 1st–3rd branch order capillaries (anesthetized mice: Fig. 1d; awake unanesthetized head-fixed mice: Extended Data Fig. 1h,i). Capillary dilation to nimodipine was largest near pericyte somata and decreased significantly with distance from the somata (Fig. 1e), consistent with pericytes mediating the nimodipine-evoked capillary dilation^12^.

Simultaneous CBF and blood pressure (BP) measurements showed that i.v. nimodipine evoked an initial fall of mean BP (presumably due to peripheral vasodilation) from 88 mmHg to 67 mmHg at 7.5 min after injection (24% drop, *P* = 0.03, paired *t*-test; Fig. 1f), which lasted approximately 30 min. This was associated with a small initial fall of CBF, but, as autoregulation mechanisms restored the BP toward its original value, the CBF rose to approximately 25% above its pre-nimodipine value. These data imply that the decrease of local vascular resistance that nimodipine evokes is larger than the decrease it evokes in systemic BP (a BP reduction with a CBF increase was also reported with another CaV blocker^22^).

Because Ca^2+^ influx through CaVs drives pericyte contraction (Extended Data Fig. 1b–g), we speculated that a mural cell [Ca^2+^]_i_ decrease would precede the nimodipine-evoked CBF increase. Using laser Doppler flowmetry and two-photon imaging of vessel diameter and mural cell [Ca^2+^]_i_ in NG2-Cre^ERT2^-GCaMP5g mice^14^, we confirmed that a nimodipine-evoked [Ca^2+^]_i_ decrease (14% in 1st–3rd order pericyte somata, 32% in 1st–3rd order pericyte processes and 22% in SMCs) preceded CBF increases and was associated with vasodilation (Fig. 1g,h,j): the mean time to half the peak [Ca^2+^]_i_ decrease in pericyte somata (1.35 ± 0.46 min) and the mean time to half the peak CBF increase at 25 min (8.41 ± 1.42 min) were significantly different (*P* = 0.004 by unpaired *t*-test; Fig. 1i). Thus, CaVs in SMCs and 1st–3rd order pericytes confer myogenic tone in WT mice.

### CaVs enhance pericyte contraction across the capillary bed

**Fig. 2:**
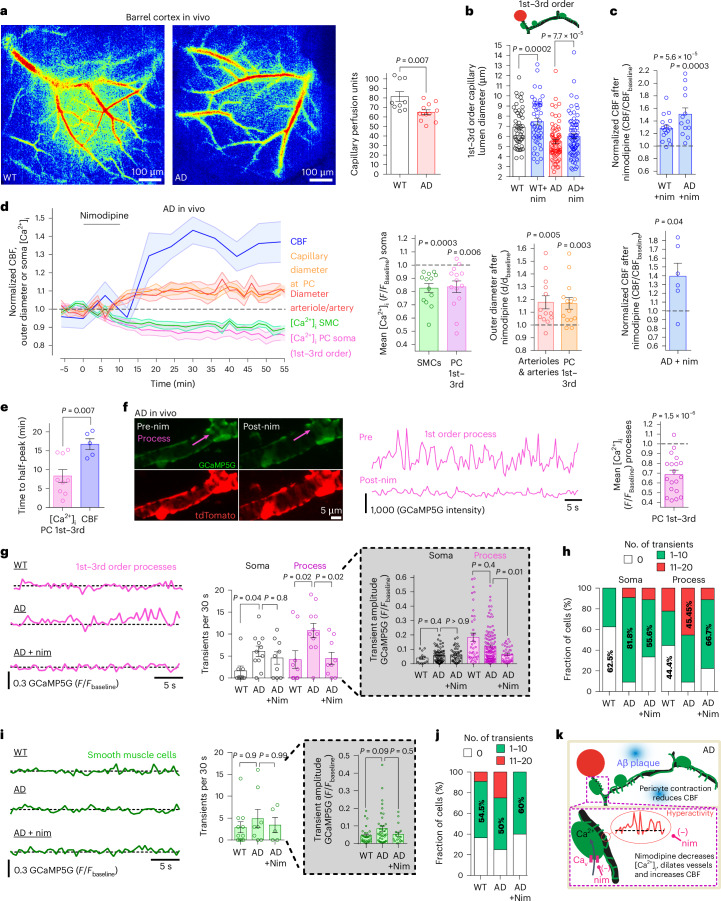
Pericyte-mediated capillary constriction and CBF decrease involve CaVs. **a**, Laser speckle imaging in the barrel cortex of WT and AD mice; blue/red show low/high perfusion. Capillary perfusion (between large vessels) was reduced in AD. *P* value: unpaired *t*-test. **b**, Capillary diameters 0–5 µm from soma center of 1st–3rd order pericytes in vivo in WT or AD NG2-dsRed mice (FITC-dextran in blood). *P* values: WT versus WT+nim paired *t*-test, AD versus AD+nim Wilcoxon test. WT data are from Fig. 1. **c**, Nim raises CBF (laser Doppler) in mature WT and AD (NG2-dsRed) mice. WT data are from Fig. 1c. **d**, Left, mean (±s.e.m.) normalized CBF, capillary diameter and pericyte soma [Ca^2+^]_i_ after i.v. nim in mature AD NG2-Cre^ERT2^-GCaMP5G mice. Bar graphs: nim reduces [Ca^2+^]_i_ in 1st–3rd order pericytes and SMCs, evoking dilation and raising CBF. Bars include some data acquired before and after nim without the whole timecourse being defined. **e**, Time to half-peak change of [Ca^2+^]_i_ or CBF in **d**. *P* value: unpaired *t*-test. **f**, Nim reduces [Ca^2+^]_i_ (image; also shows dilation) and its fluctuations (middle: pre-nim trace starts 3,004 units above zero) in processes of 1st–3rd order pericytes (AD cortex in vivo: see Supplementary Video 2). Right bar graph: mean [Ca^2+^]_i_ in processes in nim relative to baseline. *P* values in **c**,**d**,**f** from paired *t*-tests. **g**,**h**, Quantification of Ca^2+^ transients in 1st–3rd order pericyte processes and somata (2-Hz acquisition). **g**, [Ca^2+^]_i_ traces in processes of WT and AD mice with and without nim. Bar charts: transient rates and amplitudes in somata and processes. **h**, Fraction of cell somata or processes with transient rates/30 s in different ranges. **i**,**j**, As **g**,**h** but for SMCs. **k**, Summary: Aβ accumulation raises Ca^2+^ transient rates and mean [Ca^2+^]_i_, evoking pericyte contraction, capillary constriction and reduced CBF; nim reverses these changes. *P* values in **g** (soma rate and amplitudes) and **i** are from Kruskal–Wallis test with Dunn’s multiple comparison test and in **g** (process rate) from one-way ANOVA with Dunnett’s multiple comparison test. *P* values are two-tailed. Error bars are s.e.m. nim, nimodipine. Source data

CBF decreases^3^ early in human AD^10^, consistent with pericytes constricting capillaries^23,24^ (because, in a mouse AD model, capillaries are constricted, but arterioles and venules are not^12^). We, therefore, tested whether CaVs contribute to the pericyte-mediated capillary constriction reported in the NG2-dsRedxAPP^NL-G-F^ mouse model of AD^12^, which has Swedish, Arctic and Iberian mutations of APP knocked-in to avoid APP overexpression artifacts^25^ and has mural cells labeled with dsRed. From hereon, we refer to APP^NL–G–F^ mice as AD mice. Consistent with the CBF decrease in other models of AD^26–29^, we found, using laser speckle imaging (Methods), that capillary perfusion was significantly reduced in the barrel cortex of P191–P206 AD mice compared to WT mice (Fig. 2a). In vivo two-photon imaging of 1st–3rd order capillaries in eight mature P125–P158 AD mice revealed a narrowing of the capillary lumen of 21% at pericyte somata compared to age-matched P120–P158 WT mice (*P* = 3.0 × 10^−5^, Mann–Whitney test; Fig. 2b). A similar capillary constriction is seen in other AD mouse models^30–33^ and may be driven by Aβ-evoked ROS and ET-1 release contracting pericytes^12,32^.

Treating mature AD mice with i.v. nimodipine increased the capillary diameter at 1st–3rd order pericyte somata by 9.6% to 6 µm (*P* = 7.7 × 10^−5^, Wilcoxon test), that is toward the 6.9-µm capillary diameter in 1st–3rd order WT mice (Fig. 2b), and raised CBF (from laser Doppler flowmetry) by 51% (*P* = 0.0003; Fig. 2c). To test whether the nimodipine-evoked vasodilation increases CBF by reducing mural cell [Ca^2+^]_i_, we combined laser Doppler flowmetry with two-photon imaging in mature ADxNG2-Cre^ERT2^-GCaMP5g mice. As in WT mice (Fig. 1h), in these AD mice, the nimodipine-evoked drop in mural cell somatic [Ca^2+^]_i_ was associated with increases in capillary and arteriole diameters and a slower 35% rise in CBF (Fig. 2d): the mean time to half the peak [Ca^2+^]_i_ decrease in pericyte somata (8.33 ± 1.70 min) and the mean time to half the peak CBF increase at 36 min (16.7 ± 1.45 min) differed significantly (*P* = 0.007, unpaired *t*-test; Fig. 2e). Nimodipine significantly reduced mean [Ca^2+^]_i_ in 1st–3rd order pericyte processes of AD mice (Fig. 2f and Supplementary Video 2), which covered capillaries to a similar extent as in WT mice (Extended Data Fig. 2a).

For the simultaneously recorded diameter and blood flow timecourse data in the bar charts of Fig. 2d, the arterioles and capillaries (at pericytes) increased in outer diameter (which slightly underestimates the change in lumen diameter) by 17.8% and 16.9%, respectively, in response to nimodipine, and blood flow was increased by approximately 40%. To assess whether the nimodipine-evoked increase of capillary and arteriole diameters could account for the observed increase of CBF, we used a previous mathematical model^12^, with 43% of the vascular bed resistance in arterioles (where resistance is inversely proportional to the 4th power of diameter) and 57% in capillaries (where resistance depends on the spatial profile of diameter around pericytes^12^). After an AD-evoked decrease of capillary diameter at pericytes by 21% (Fig. 2b) with no change of arteriole or venule diameter^12^, the model predicted that the nimodipine-evoked 16.9% increase of the diameter of capillaries (at pericytes) and the 17.8% increase for arterioles (Fig. 2d) would increase CBF by 51%, similar to the 40–51% increase observed with laser Doppler measurements (Fig. 2c,d). We conclude that the effect of nimodipine on CBF is explained by the increases of vessel diameter that it evokes, without needing to invoke effects of nimodipine on cells other than pericytes and SMCs.

In 1st–3rd branch order pericytes, [Ca^2+^]_i_ transient frequency was increased 3.7-fold in somata and 2.6-fold in processes in AD mice (Fig. 2g,h), whereas there was no significant change in pericyte process [Ca^2+^]_i_ transient amplitude (Fig. 2g) or SMC [Ca^2+^]_i_ transient frequency or amplitude (Fig. 2i,j). Treating AD mice with nimodipine largely restored 1st–3rd order [Ca^2+^]_i_ transient frequency in pericyte processes to WT levels (Fig. 2g) and reduced the transient amplitude (Fig. 2g, inset). Vehicle treatment did not significantly modulate SMC or pericyte [Ca^2+^]_i_ nor the diameter of arteries, arterioles or capillaries (Extended Data Fig. 2d). Repetitive imaging with 800-nm light (for which GCaMP5G fluorescence lacks Ca^2+^sensitivity) evoked a constant fluorescence with no visible transients or photobleaching decay (Extended Data Fig. 2e–g and Supplementary Video 3), suggesting that photobleaching, motion artifacts and preparation rundown did not influence measured [Ca^2+^]_i_. Nimodipine thus reduces the fluctuations as well as the mean [Ca^2+^]_i_ in 1st–3rd order pericytes. Comparing the nimodipine-evoked CBF increase between 19 mature WT and 11 mature AD mice (P110–P191) across all genotypes (regardless of dsRed (Fig. 2c) or NG2-Cre^ERT2^-GCaMP5g (Fig. 2d) co-expression), the CBF increase was 74% larger in AD mice (47% rise) than in WT mice (27% rise) (*P* = 0.03, unpaired *t*-test with Welch’s correction; Extended Data Fig. 2c), implying greater CaV-evoked tone in the AD mice (as expected from pericyte-mediated capillary constriction occurring in AD^12^ and the contribution of CaVs to generating pericyte tone^14^). Thus, block of CaVs in AD, for example with nimodipine, is a potential route to reversing the decrease of CBF that occurs early in AD (Fig. 2k).

**Fig. 3:**
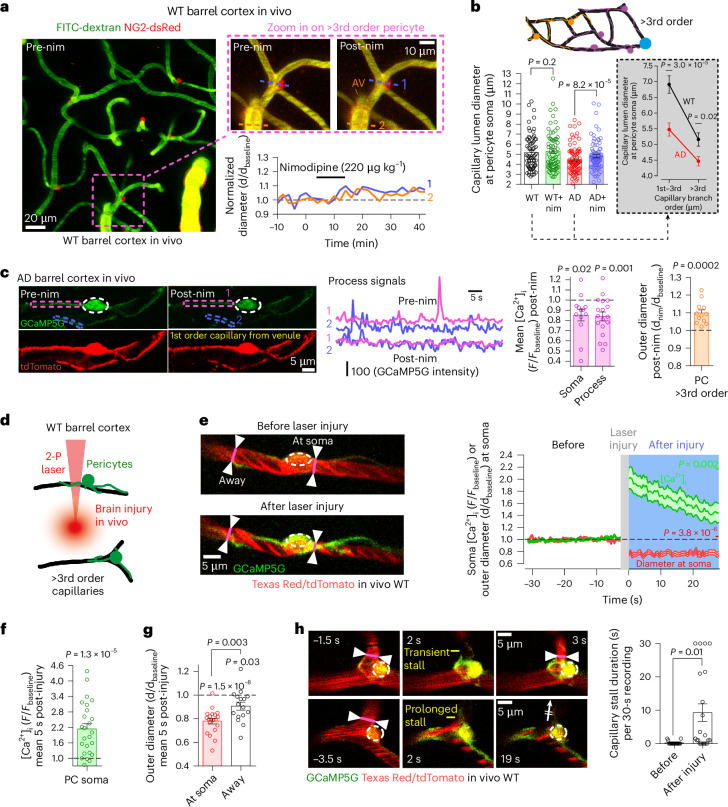
Mid-capillary bed pericytes exert enhanced contractile tone in AD mice. **a**, In vivo two-photon imaging of mid-capillary bed in the barrel cortex of a WT NG2-dsRed mouse with FITC-dextran in the blood. Lines in the inset show sites of diameter measurements (graph: nim has little effect in WT vessels) at a pericyte soma (red, 1) on a capillary of >3rd branch order from the nearest PA and 2nd branch order from the AV, and at the AV (2). **b**, Intravenous nim partially restores capillary diameter decreases at the soma of >3rd order pericytes in AD NG2-dsRed mice. Inset: capillary diameter as a function of branch order in WT and AD mice in the absence of nim. *P* values for WT versus WT+nim (pre-nim versus post-nim) and AD versus AD+nim are from (paired) Wilcoxon tests; those for WT versus AD are from unpaired Mann–Whitney tests. **c**, Nim reduces [Ca^2+^]_i_ in the soma and processes of >3rd order pericytes in vivo in AD NG2-Cre^ERT2^-GCaMP5G mice (images, colored traces are for processes 1 and 2 on images; pre-nim traces start at 368 and 310 for 1 and 2; left bar graph shows mean normalized changes). Nim evokes capillary dilation at pericyte somata (right bar graph). **d**, Schematic of laser-evoked in vivo brain injury near >3rd order capillaries in the barrel cortex of WT NG2-Cre^ERT2^-GCaMP5G mice. **e**–**g**, Laser-evoked injury raises [Ca^2+^]_i_ (**e**,**f**) and contracts >3rd order pericytes near their somata (**e**,**g**; see also Supplementary Video 4 and Extended Data Fig. 4b). Injury-evoked capillary constriction was attenuated away from pericyte somata (**g**, *P* values above bars compare bar means with unity). **h**, Brain injury induces capillary stalling of blood near pericyte somata. Transient and prolonged capillary blocks were seen as in top and bottom panels. *P* values are from paired *t*-tests in **c**,**f**, paired and unpaired *t*-tests in **g** and a Wilcoxon test in **e**,**h**. *P* values are two-tailed. Error bars are s.e.m. 2-P, two-photon; nim, nimodipine. Source data

**Extended Data Fig. 4:**
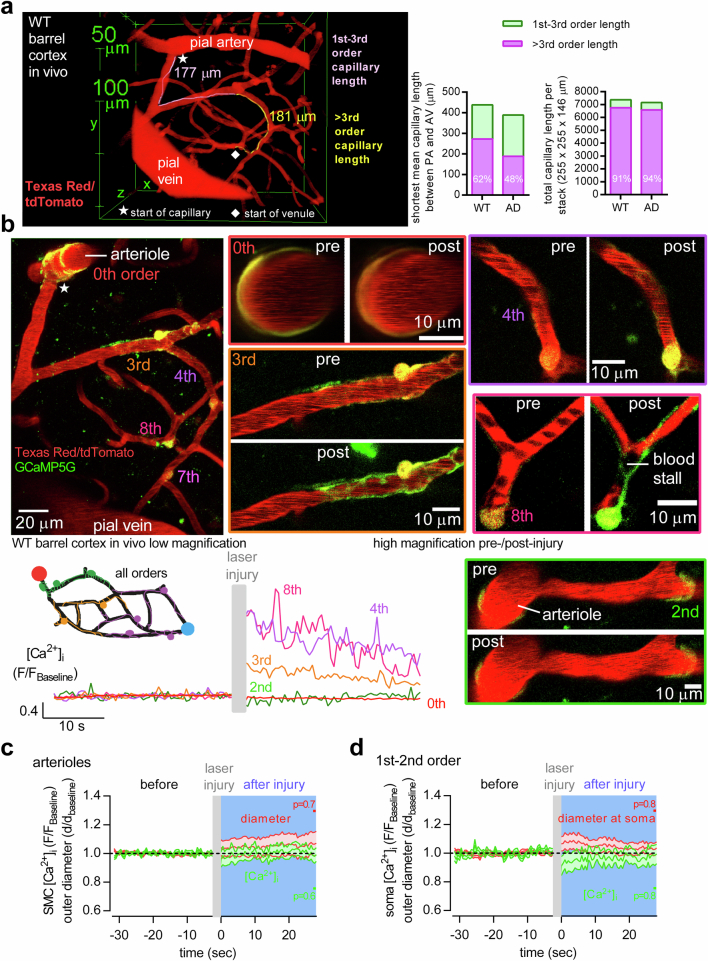
Capillaries in the middle and on the venule side of the capillary bed are a major vascular compartment, covered by pericytes that generate contractile tone in response to laser injury (unlike pericytes and SMCs on the arteriole side of the vascular bed). **a** 3D capillary tracing of barrel cortex microvasculature in vivo from the start of the 1^st^ capillary branch off a penetrating arteriole (star, also shown in (b) as a z projection without the overlying pial artery; note that in (a) the arteriole is hidden behind the artery) to the start of the venule (diamond) connecting to a pial vein in an NG2-Cre^ERT2^-GCaMP5g WT mouse (with Texas Red given i.v.). Left bar graph: shortest capillary length between PA and AV. Right bar graph: capillaries of the >3^rd^ branch order comprise >90% of the total capillary length in WT and AD mice. **b** Left: low magnification in vivo two-photon z projection of capillaries branching from arteriole (star marking start of 1^st^ capillary branch as shown in 3D in (a)) in WT mouse. Branching orders in high magnification images on the right and in the bottom [Ca^2+^]_i_ traces are indicated as numbers; these images are color-framed (according to position in vascular bed, see inset) and shown pre- and post-laser injury. Bottom traces: laser injury evoked [Ca^2+^]_i_ rises increase with increasing vessel branching order (see also Fig. 3d–h and Supplementary Video 4). The 7th order capillary in the low power view is the same capillary rotated in Fig. 3e. **c-d** Laser injury does not significantly modulate [Ca^2+^]_i_ or diameter of arterioles (**c**) or 1^st^-2^nd^ order capillaries (**d**) in WT mice (see also Supplementary Video 5). P-values are 2-tailed. Error bars are s.e.m. Source data

Although contractile pericytes on 1st–3rd order capillaries (as above) can rapidly adjust capillary diameter^34^, we found that capillaries in the middle and on the venule side of the capillary bed, referred to hereafter as ‘>3rd order’, comprise 91% of the total capillary length in WT mice and 94% in AD mice (Extended Data Fig. 4a). Because >3rd order pericytes may generate contractile tone over a slow timescale^15,17^ and occupy most of the capillary bed (where most of the brain’s vascular resistance lies^35^), they may exert a strong influence on CBF in response to nimodipine. However, measuring >3rd order capillary diameters at pericyte somata in WT mice in vivo showed no significant change in capillary diameter after i.v. nimodipine treatment (Fig. 3a,b). The diameter of venules and veins was also not significantly changed by nimodipine in WT mice (increased by 12.2%, paired *t*-test, at t = 40 min, paired *t*-test, *P* = 0.06, *n* = 7). In contrast, in AD mice, >3rd order capillaries were significantly more constricted (by 13.5%) at pericyte somata compared to WT mice, and treatment with i.v. nimodipine partially reversed this constriction (Fig. 3b), consistent with nimodipine reducing [Ca^2+^]_i_ in >3rd order pericyte somata and processes (Fig. 3c). Notably, even though the [Ca^2+^]_i_ transient frequency was elevated in >3rd order pericyte somata of AD compared to WT mice, nimodipine did not modulate the [Ca^2+^]_i_ transient frequency in the soma or processes of >3rd order pericytes, but it did decrease the amplitude of the transients (Extended Data Fig. 2h).

The ability of >3rd order pericytes to adjust capillary diameter in response to ischemia insults has been disputed^36^. To test whether >3rd order pericytes can contract (as may occur in ischemic and AD cortex where levels of vasoconstrictors, such as ROS, are elevated^37,38^), we induced two-photon laser parenchymal injuries generating ROS^39^, with the laser beam targeted to approximately 15–20 µm away from single capillaries in the cortex of NG2-Cre^ERT2^-GCaMP5g WT mice (Fig. 3d). We detected strong [Ca^2+^]_i_ rises in 3rd or higher order pericytes, which coincided with capillary constriction at pericyte somata, but significantly less constriction at parts of the capillary lumen ≥20 μm away from those somata (Fig. 3e–g, Extended Data Fig. 4b and Supplementary Video 4). Laser-evoked pericyte contraction also strongly enhanced the stalling of blood, which appeared as shadows in the dye-filled plasma (Fig. 3h). Surprisingly, identical laser-evoked insults near 1st–2nd order capillaries or arterioles did not modulate vessel diameter or [Ca^2+^]_i_ in pericytes or SMCs (Extended Data Fig. 4b–d and Supplementary Video 5). Although mural cell responses to local laser-evoked injury thus vary along the vascular tree, these data confirm that >3rd order pericytes can generate capillary constriction^15,17^.

### Immune cell ROS raise [Ca^2+^]_i_ and reduce CBF in AD mice

**Fig. 4:**
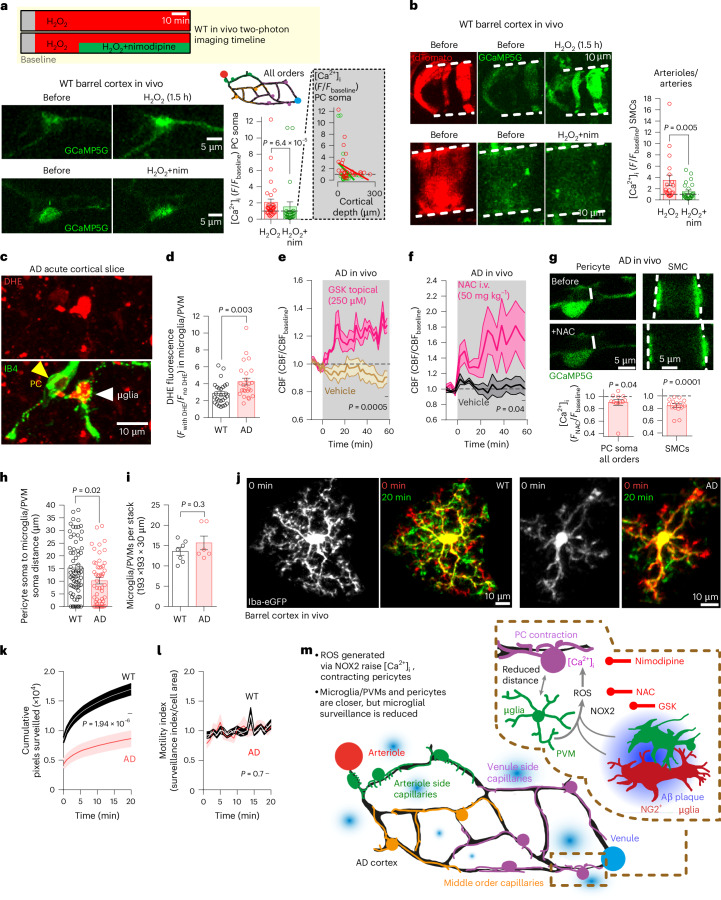
ROS from brain immune cells drive pericyte contraction in AD mice. **a**, In vivo two-photon imaging of pericytes in the barrel cortex of NG2-Cre^ERT2^-GCaMP5G mice before and after H_2_O_2_ application without or with nim (see timeline). Inset: H_2_O_2_-evoked [Ca^2+^]_i_ rise versus cortical depth. **b**, As **a** but for SMCs of pial arteries and PAs (green signal is [Ca^2+^]_i_; red is td-Tomato expressed with GCaMP5g). **c**, Confocal image of parenchymal microglial (μglia) cell generating ROS (DHE) in acute cortical slice of AD mouse (microglia and pericytes (PCs) labeled using IB4). **d**, Microglia generate more ROS in AD than in WT. **e**, In AD mice, NOX2 block with GSK2795039 (but not vehicle) increases CBF. **f**,**g**, The antioxidant NAC (but not vehicle) increases CBF (**f**) and lowers [Ca^2+^]_i_ in pericyte somata and SMCs (**g**). **h**, Cortical microglia/PVMs are closer to pericytes in AD mice than in WT mice (assessed as distance of NG2-dsRed-labeled pericyte soma surface to Iba1-stained cell surface). **i**, Microglia/PVM numbers do not differ in the barrel cortex of mature WT and AD mice. **j**, In vivo two-photon imaging of microglia in WT and ADxIba1-eGFP mice. Overlay of 0 min (red) and 20 min (green) images shows that microglia extend and retract processes over time. **k**, Cumulative number of pixels surveilled by 21 WT and 16 AD microglia in maximum intensity projection images as in **j**. Initial value is cell area at t = 0. **l**, Microglial motility index (ratio of surveillance index to cell area) is unaltered in AD mice. **m**, Pathway evoking capillary constriction in AD. Lower left, vascular schematic with Aβ plaques (blue) surrounded (top right) by NG2^−^ (green) and NG2^+^ (red) microglia. Top right, ROS generated by microglia and NG2-expressing microglia raise PC [Ca^2+^]_i_ and evoke contraction. Targeting the pathway by blocking CaVs with nim or ROS with NAC or GSK improves CBF. *P* values are from Mann–Whitney tests in **a**–**d**,**f**,**h**, unpaired *t*-tests in **e**,**i**,**k**,**l** and a Wilcoxon test and paired *t*-test in **g** for PCs and SMCs, respectively. *P* values are two-tailed. Error bars are s.e.m. nim, nimodipine. Source data

The above data show that pericyte CaVs mediate capillary constriction throughout the capillary bed in the AD cortex in vivo, but the constriction mechanism is poorly understood. Aβ oligomers, when applied acutely to WT brain slices, generate ROS, which release ET-1 and, thus, evoke contraction by activating CaV channels^12,14^. Similarly, oxidative stress occurs in human AD^37^ and impairs CBF^40,41^ in AD models. Performing in vivo two-photon imaging in NG2-Cre^ERT2^-GCaMP5g mice (Fig. 4a), we found that generating oxidative stress, by applying 1 mM H_2_O_2_ topically to the exposed barrel cortex, increased pericyte [Ca^2+^]_i_ 2.07-fold (Fig. 4a), consistent with reports of ROS evoking pericyte contraction^12,38^. The H_2_O_2_-evoked [Ca^2+^]_i_ rise decreased with cortical depth (Fig. 4a, inset), suggesting that H_2_O_2_ diffusion is limited to the upper cortical layers. For pericytes of matched cortical depth, nimodipine (220 μg kg^−1^ i.v.) reduced the H_2_O_2_-evoked [Ca^2+^]_i_ rise by 54% to 1.49-fold (Fig. 4a), so CaVs mediate at least half of this [Ca^2+^]_i_ rise in vivo. Nimodipine also greatly reduced the H_2_O_2_-evoked [Ca^2+^]_i_ rise in SMCs (Fig. 4b).

**Extended Data Fig. 5:**
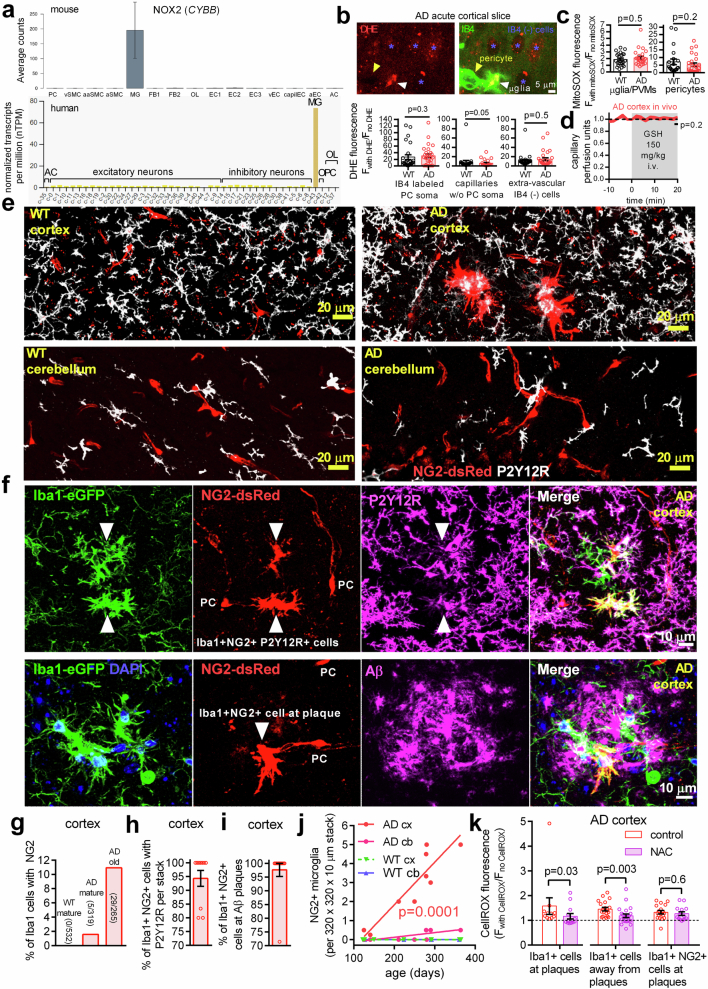
Brain immune cells generate ROS in AD. **a** scRNA sequencing in mouse cortex and human brain: the major cerebral ROS-generating NADPH oxidase, NOX2, is only expressed in microglia/PVMs. Upper graph abbreviations: mRNA level (counts), pericytes (PCs), venous SMCs (vSMCs), arteriolar SMCs (aSMCs), arterial SMCs (aaSMCs), microglia (MG), fibroblast classes (FB1, FB2), oligodendrocytes (OL), broadly-distributed endothelial cell classes (EC1-EC3), venous ECs (vEC), capillary ECs (capilEC), arterial ECs (aEC), astrocytes (AC), from^43^. Lower graph: 45 clusters (c-0 to c-44) of neuronal and glial cells including MG, ACs, oligodendrocyte progenitor cells (OPCs) and subtypes of excitatory neurons, inhibitory neuons and OLs, from ref. ^94^ (v23.proteinatlas.org). **b** Top: confocal images of live AD acute cortical slice with DHE-labeling of ROS in microglia (white arrow) but not pericytes (yellow arrow) or extravascular cells (blue asterisks). Bottom: DHE labeling quantification in WT and AD cortical slices in pericyte somata, capillary segments without (w/o) pericyte somata and (non-immune) extra-vascular cells. **c** Mitochondrial ROS measured using MitoSOX in microglia or pericyte somata does not differ in WT and AD slices. P values: b-c from Mann-Whitney tests,d from paired t-test. **d** Laser speckle imaging in barrel cortex of AD mice in vivo shows intravenous glutathione (GSH) does not alter capillary perfusion. **e** NG2-expressing microglia are in cortex but not cerebellum of mature AD mice nor in cortex and cerebellum of WT mice. **f** Top: NG2-expressing microglia (arrow heads) but not pericytes (PCs) express Iba1 and P2Y12Rs. Bottom: NG2-expressing microglia cluster around Aβ plaques (labeled with 82E1 antibody). **g** Percentage of Iba1 cells co-labeling for NG2. **h** Most Iba1-NG2-labeled cells co-express P2Y12Rs. **i** Most NG2-expressing microglia are at Aβ plaques. **j** Number of NG2-expressing microglia increases with ageing in AD cortex. P-value for AD cortex (cx) graph assesses whether the slope of linear regression fit deviates from 0 (cb=cerebellum). **k** ROS generation measured using CellROX is increased in immune cells at and away from plaques in AD cortex. The antioxidant NAC reduces ROS production. P values are from Mann-Whitney tests for Iba1+ cells and unpaired t-tests for NG2-expressing microglia. P-values are 2-tailed. Error bars are s.e.m. Source data

**Extended Data Fig. 6:**
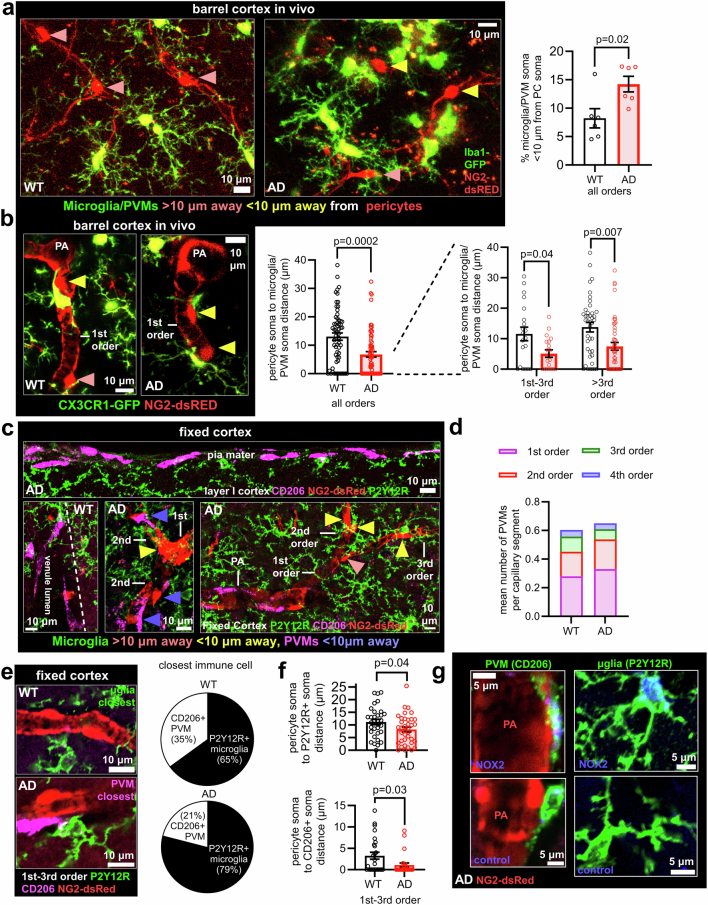
Microglial and perivascular macrophage (PVM) interactions with pericytes are enhanced in AD mice. **a** In vivo two-photon imaging in the barrel cortex of WT and AD x Iba1-eGFP x NG2-dsRed mice used to quantify the percentage of microglia/PVM somata <10 µm vs. >10 µm from the nearest pericyte soma. Yellow and light red triangles point at pericytes that are <10 and >10 µm from microglia/PVMs, respectively. **b** Microglia/PVMs are located closer to 1^st^-3^rd^ order and >3^rd^ order pericytes in AD mice (co-expressing NG2-dsRed either with Iba1-eGFP or CX3CR1-eGFP) than in WT mice. **c** CD206-labeled PVMs in the pia mater (top panel), along venules and arterioles (bottom left and right panel), and on capillaries of the 1^st^-2^nd^ branch order off an arteriole (bottom middle and right panels). Blue triangles point at pericytes that are <10 µm from PVMs. Yellow and light red triangles point at pericytes that are <10 and >10 µm from P2Y12R-labeled microglia, respectively. **d** Mean number of PVMs per capillary segment decreases as the branch order from the arteriole increases (quantified from images as shown in c). **e** Percentage of 1^st^-3^rd^ order pericytes closest to a CD206 or P2Y12R-labeled cell in fixed cortical tissue from WT and AD mice. **f** Distance of 1^st^-3^rd^ order pericyte soma to P2Y12R or CD206-expressing soma quantified from images such as shown in e. **g** Fixed AD cortical tissue imaging of Alexa Fluor 647 conjugated NOX2 antibody (or 647-excited autofluorescence control) in microglia and PVMs. P values in panels **a** and upper bar graph of f are from unpaired t tests and in b and lower bar graph of f from Mann-Whitney tests. P-values are 2-tailed. Error bars are s.e.m. Source data

Pericyte contraction evoked by acutely applying Aβ depends partly on microglial and perivascular macrophage (PVM) production of ROS via NOX2, which, in the brain, including in human AD, is expressed in microglia and PVMs (Extended Data Figs. 5a and 6g)^12,42–44^. The contribution of microglia and PVMs may vary with position in the capillary bed, because PVMs are more numerous around arterioles and the first few branches of the capillary bed (Extended Data Fig. 6c,d). To test whether Aβ accumulation in AD similarly produces ROS in microglia, we incubated acute cortical slices of WT and AD mice with the ROS sensor dihydroethidium (DHE), which fluoresces when oxidized DHE intercalates into DNA. In isolectin B4-labeled microglia/PVMs, DHE fluorescence (normalized to the autofluorescence in the absence of DHE) was increased by 49% in AD mice compared to WT mice (Fig. 4c,d), despite the autofluorescence being higher in the AD mice. Similarly, ROS production by microglia/PVMs in AD was confirmed using the ROS sensor CellROX in cortical slices from ADxNG2-dsRed mice expressing enhanced green fluorescent protein (eGFP) under the monocyte lineage promotor Iba1 (Extended Data Fig. 5k; see below). In contrast, no change in DHE fluorescence was detected in pericytes nor in other vascular cells (that were not IB4-labeled pericytes) or extravascular cells other than microglia/PVMs (defined as IB4-negative cells appearing as shadows in the extravascular space of IB4-labeled cortical slices; Extended Data Fig. 5b). Furthermore, mitochondrial ROS measured using MitoSOX in microglia or pericytes did not differ between WT mice and AD mice (Extended Data Fig. 5c).

A rise in ROS level could contribute to the CBF decrease in AD by triggering Ca^2+^ entry through mural cell CaVs (Fig. 4a,b), so we tested whether CBF in AD mice can be modulated by blocking microglial/PVM production of ROS via NOX2. Local application of the NOX2 blocker GSK2795039 (GSK) to the cortical surface of AD mice in vivo increased CBF by 42% (compared to vehicle treatment; Fig. 4e). A similar improvement in capillary flow was reported with other NOX2 blockers in AD mouse models^40,41^. To better mimic a clinical scenario, we therefore tested how a clinically approved ROS scavenger, the BBB-permeable antioxidant N-acetyl cysteine (NAC), modulates CBF in AD mice. Treatment with NAC (50 mg kg^−1^ i.v.) increased CBF by 62% (Fig. 4f) and lowered [Ca^2+^]_i_ in pericyte somata and SMCs (Fig. 4g), consistent with NAC reducing ROS-evoked [Ca^2+^]_i_ rises in mural cells^39^ and blocking their contraction^15^. In contrast, treatment with the antioxidant glutathione (150 mg kg^−1^ i.v.), which, unlike NAC, poorly crosses the BBB^45^ (and, thus, predominantly scavenges peripheral ROS), did not modulate CBF (Extended Data Fig. 5d).

Because Aβ plaques are a reservoir for Aβ oligomers^46^ and ROS^47^, driving a distinct inflammatory profile in microglia^48^, we investigated whether ROS production differs in microglia at (<65 μm from) versus away from (>65 μm from) Aβ plaques and whether ROS can be targeted by NAC. Using the ROS sensor CellROX, we measured ROS in cortical slices from ADxNG2-dsRedxIba1-eGFP mice. ROS were measured in (1) Iba-1-expressing microglia at plaques, (2) Iba1-expressing microglia away from plaques and (3) plaque-associated microglia that co-express Iba1 and P2Y_12_ receptors (P2Y_12_Rs) (commonly used as microglial labels) with the pericyte and oligodendrocyte precursor cell (OPC) marker NG2 (Extended Data Fig. 5e–h). This third cell class was previously noted in facial nerve axotomy, ischemia, Parkinson’s disease and AD, and transcriptomic characterization revealed that these cells are highly proliferative^49–51^. NG2-expressing microglia were mainly found at plaques (Extended Data Fig. 5f,i) and increased in number with aging in AD cortex (where plaque burden worsens with age) but were absent in WT mouse brains and only appeared late in AD cerebellum (Extended Data Fig. 5j) where Aβ deposits appear very late in AD and which exhibits no change in CBF^52^. Adding CellROX increased the fluorescence at the 633-nm wavelength (which, in the absence of CellROX, is autofluorescence) in the cortex by 57% in Iba1-labeled microglia at plaques, by 45% in Iba1-labeled microglia away from plaques and by 33% in NG2-expressing microglia (Extended Data Fig. 5k). Pre-incubation with 50 µM NAC significantly reduced the CellROX-evoked fluorescence increases to 16% and 18% at microglia at and away from plaques, respectively, but did not significantly reduce the CellROX fluorescence in NG2-expressing microglia (Extended Data Fig. 5k). ROS production was, thus, largely blocked by NAC in AD mice.

Consistent with microglia migrating toward vessels in AD and during brain inflammation^53,54^, we found that the distance between Iba1-labeled microglia/PVM somata and pericyte somata was reduced in the barrel cortex in vivo and in the cortex of perfusion-fixed ADxNG2-dsRedxIba-eGFP mice compared to in WT mice (Fig. 4h and Extended Data Fig. 6a,b). This was true for pericytes on both 1st–3rd and >3rd branch order capillaries (Extended Data Fig. 6b): thus, microglia/PVM somata are closer to pericyte somata throughout the capillary bed in AD mice. Pericyte densities per capillary length (Extended Data Fig. 2b), microglial density^53^ (Fig. 4i), PVM density (PVM number per capillary segment on 1st–4th order capillaries in fixed WT and AD cortex, *P* = 0.9, Mann–Whitney test; see also Extended Data Fig. 6c,d) and brain atrophy (see below) were the same in 4–6-month-old WT and AD mice, ruling out changes in these parameters as causing the closer proximity of pericytes to microglia in AD. Interestingly, the proportion of microglia less than 10 μm from pericytes in the superior frontal gyrus was reported to decrease^55^ in human AD, but, in our mice, the proportion of microglia somata within 10 μm of pericyte somata was increased in AD (Extended Data Fig. 6a). Only approximately 4% of microglia were less than 10 μm from pericytes in the human study^55^, so the mean pericyte–microglial cell distance would be dominated by the 96% of microglia that were not ‘pericyte associated’. The causes of these differences with the human paper^55^ are uncertain, but they may reflect the humans having more advanced AD (than our mice), which is associated with pericyte loss (unlike in our study: Extended Data Fig. 2b), or that a different brain area was studied. Consistent with enhanced interaction of Iba1-expressing cells with pericytes in AD (Fig. 4h), we found that the distance between P2Y12R-labeled microglial somata or CD206-labeled PVM somata and 1st–3rd branch order pericyte somata was reduced by 27% and 67%, respectively, in ADxNG2-dsRed mice compared to WT mice (Extended Data Fig. 6f).

In vivo imaging of the barrel cortex in WT and ADxIba1-eGFP mice revealed that cumulative pixel surveillance by microglia (over 20 min) was greatly reduced in AD mice compared to WT mice (Fig. 4j,k). However, there was no change in cell-size-independent motility (the surveillance index of each cell divided by its area) in AD mice (Fig. 4l), suggesting that the reduced surveillance reflects microglia being less ramified in AD mice than in WT mice (Fig. 4j). Thus, although surveillance decreases could reduce interactions of microglial processes with capillaries in AD, microglial/PVM somata are in closer proximity to blood vessels in AD mice compared to WT mice, which may shorten the distance over which released ROS need to diffuse to modulate vascular tone. Figure 4m summarizes how impaired vascular–immune cell interactions may evoke pericyte contraction in AD.

### Venule-end pericytes drive leukocyte stalling in AD mice

**Fig. 5:**
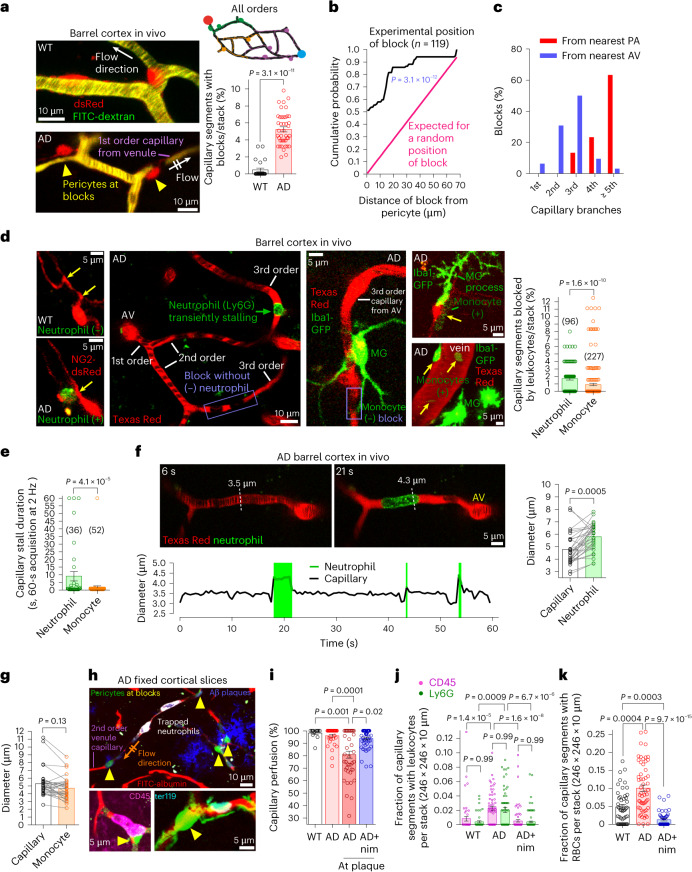
CaV inhibition reduces pericyte-evoked capillary block by blood cells in AD. **a**, Imaging cortex of NG2-dsRed WT and AD mice; FITC-dextran in blood. Yellow triangles: pericytes at blocks (Supplementary Video 6). Plot: percentage of capillary segments with blocks in 94 μm × 94 μm × 10 μm image stacks. **b**, Probability distribution of distance of nearest pericyte soma to block (black) in AD cortex. Magenta: prediction for pericytes uniformly spaced along capillary (Extended Data Fig. 2b) if blocks occur randomly (*P* value: Kolmogorov–Smirnov test). **c**, Percentage of blocks in different branch order capillaries from PA or AV in AD. **d**, In vivo imaging of Ly6G-labeled neutrophils or Iba1-eGFP-expressing monocytes in lumen (labeled with Texas Red or (left-most and top right panels) outlined by NG2-dsRed pericyte processes). Yellow arrows: left, pericyte circumferential processes; right, PVMs and monocytes (top right, monocyte also indicated by a green line). Blue boxes: blocks without neutrophils or monocytes. Graph: percentage of capillaries with blocks containing neutrophil or monocyte (Supplementary Videos 7 and 8). **e**, Stall duration of cells in AD cortex. **f**, Capillary images and diameters during neutrophil stall in AD (Supplementary Video 9). Graph: stalled neutrophils are larger than capillary lumen (without a neutrophil) where they stall. **g**, Stalled monocytes are not larger than capillaries they stall in. **h**, AD mice perfused with FITC-albumin in gelatin (re-colored red) when alive (to label patent vessels) show impaired perfusion at pericyte somata (yellow triangles). Capillary blocks contain Ly6G-labeled neutrophils (top), CD45-labeled leukocytes (bottom left) and ter119-labeled RBCs (bottom right). Aβ plaques labeled with 82E1 in top image. **i**–**k**, Nimodipine in vivo largely restores capillary perfusion (percent of vessel segments that are patent) at Aβ plaques (**i**, pale pink bar is for capillaries away from plaques) and reduces the number of leukocytes (**j**) and RBCs (**k**) stuck in capillaries. Note, there may be more than one cell type per block, with RBCs also present at leukocyte blocks. *P* values are from Mann–Whitney tests in **a**,**d**–**g**, and Kruskal–Wallis tests in **i**–**k**. *P* values are two-tailed. Error bars are s.e.m. Source data

To test whether pericyte-evoked narrowing of the capillary lumen in AD (Figs. 2b,d and 3b,c) causes blood flow to stall^21^ near pericyte somata where constriction is largest^12^, we acquired three-dimensional in vivo two-photon imaging stacks capturing each capillary segment for approximately 3–5 s (in ~3–5 frames depending on capillary orientation). As previously reported^19,56,57^, we found that the percentage of capillaries blocked was increased from 0.47% in WT mice to 5.2% in AD mice (Fig. 5a and Supplementary Video 6). If blocks were randomly positioned in capillaries, then the probability of blocks occurring should be constant at distances up to half the inter-pericyte distance, which (for one pericyte per 144 µm: Extended Data Fig. 2b) is 72 µm (at larger distances, the block would be closer to the next pericyte along the capillary). Measuring the distance from 119 block sites in capillaries to the nearest pericyte soma revealed that the cumulative probability distribution for a random block position (a straight line reaching unity at 72 µm) differed signficantly from the experimentally observed one; distances between blocks and pericytes were closer than expected for randomly positioned blocks of capillaries (Fig. 5b; *P* = 3.1 × 10^−12^, Kolmogorov–Smirnov test).

Tracking capillary branch order from PAs in AD mice showed no blocks in 1st–2nd order capillaries, 13% in 3rd order capillaries and 87% in ≥4th order capillaries from PAs. Tracking the branch order from ascending venules (AVs), 87% of blocks were in 1st–3rd order capillary branches from AVs (Fig. 5c). Capillary blocks thus occur mainly at the venule end of the capillary bed where capillary diameter is smallest (and reduced further in AD; Fig. 3b).

Because circulating monocytes and neutrophils are recruited to the AD brain and are less distensible and larger than erythrocytes, we explored their contribution to capillary stalls by imaging monocytes using ADxIba1-eGFP mice and neutrophils using a low dose of fluorescently conjugated antibody against Ly6G (0.1 mg kg^−1^ i.v., which does not induce neutropenia^19^). In three-dimensional imaging stacks (as in Fig. 5a), we found that 1.7% of capillaries showed blocks containing neutrophils^19^ and 0.9% showed blocks containing monocytes (Fig. 5d). We did not label neutrophils and monocytes simultaneously (so blocks with neutrophils may also contain monocytes), but, together, these could account for half of the 5.2% of capillary blocks (Fig. 5a), with the remaining blocks possibly containing RBCs (see below). Because leukocyte stall duration determines the extent of the CBF decrease that stalls evoke, we captured the onset and offset of stalls by imaging capillaries at 2 Hz in a single plane (Supplementary Video 7). The mean stall duration for neutrophils was 5.4-fold longer than that for monocytes (Fig. 5e), possibly because they are less flexible or adhere to endothelial cells more (some monocytes also transiently adhered to pial veins or arteries without preventing blood flow (Fig. 5d, bottom right image, and Supplementary Video 8)).

**Extended Data Fig. 7:**
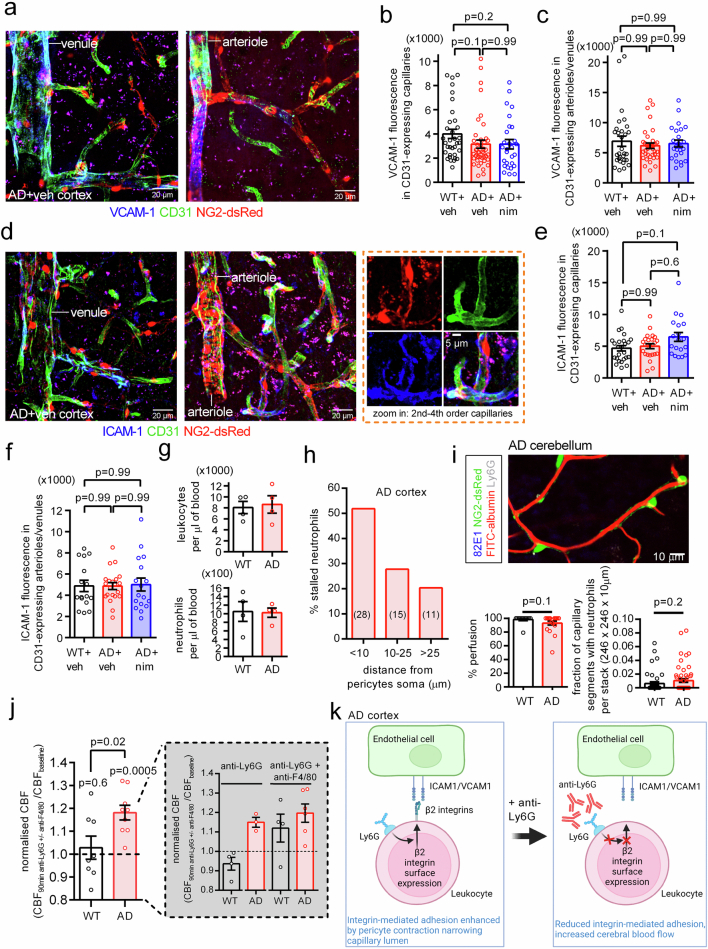
Long-term nimodipine treatment does not modulate cortical expression of VCAM-1 or ICAM-1 in endothelial cells of AD mice and blood leukocyte numbers do not differ between WT and AD mice. **a** Confocal images of VCAM-1 and endothelial CD31 staining in fixed cortical slices after ~1.5 months of treatment with the vehicle for nimodipine (DMSO) in an AD (NG2-dsRed) mouse. Capillaries branch from the ascending venule (left) and penetrating arteriole (right). **b-c** VCAM-1 fluorescence intensity measured in capillaries (**b**) or arterioles/venules (**c**) of WT mice treated with vehicle or in AD mice treated with vehicle or nimodipine in the drinking water. **d** Confocal images of ICAM-1 and CD31 staining in fixed cortical slices in an AD (NG2-dsRed) mouse. Right: high magnification image of 2^nd^-4^th^ order capillaries branching from the arteriole. **e-f** ICAM-1 fluorescence intensity in capillaries (**e**) or arterioles/venules (**f**) of WT mice treated with vehicle or AD mice treated with vehicle or nimodipine in the drinking water. **g** Circulating leukocyte and neutrophil numbers counted using flow cytometry do not differ between WT and AD mice. **h** 52% of stalled neutrophils are within 10 µm of the pericyte soma centre in AD cortex. **i** Capillary perfusion and the number of capillaries containing adhering neutrophils does not differ between WT and AD cerebellum. **j** Low-dose anti-Ly6G (0.1 mg/kg i.v.) treatment for 1.5 hrs (to reduce β2 integrin surface expression without depleting neutrophils^58^) increases cerebral blood flow in AD mice. Co-injection of anti-Ly6G with the monocyte marker F4/80 (0.1 mg/kg) results in a similar CBF increase in AD mice as anti-Ly6G injection alone. **k** Proposed mechanism. Ly6G, a glycosylphosphatidylinositol (GPI)–anchored protein, promotes β2 integrin surface expression in neutrophils required for adhesion to VCAM-1 and ICAM-1 on endothelial cells. A low dose of anti-Ly6G ligates Ly6G without inducing neutrophil death^58^ resulting in reduced integrin-mediated adhesion and increased cerebral blood flow in AD mice. P values in panels **b**, **c** and **e** are from Kruskal^−^Wallis tests, in **f** from a one-way ANOVA, in **i** from Mann-Whitney tests and in **j** from paired t-tests and an unpaired t-test. P-values are 2-tailed. Error bars are s.e.m. Source data

Consistent with pericyte-evoked capillary narrowing driving leukocyte stalling, we found that capillary diameters at sites of neutrophil stalls were significantly smaller than the neutrophil diameters, causing the neutrophils to push against the capillary walls (Fig. 5f and Supplementary Video 9), probably enhancing their interaction with ICAM-1 and VCAM-1 adhesion molecules on endothelial cells (Extended Data Fig. 7a–f). Indeed, a low dose of anti-Ly6G, which ligates Ly6G and, thereby, reduces neutrophil surface expression of integrins and, consequently, integrin binding to ICAM-1 (without inducing neutrophil death)^58^, increased CBF in AD mice but not in WT mice (Extended Data Fig. 7j,k). Unlike neutrophils, the stalling monocyte diameters were not larger than those of the capillary segments where they stalled (Fig. 5g).

If pericyte-evoked capillary constriction drives leukocyte stalling, then increasing capillary diameter using nimodipine should relieve capillary blocks. We tested this using the FITC-albumin perfusion protocol^59^ to visualize all vessels that had been patent in vivo in fixed cortical slices, along with adhering cells that contributed to blocking the non-patent capillaries (Methods). In vehicle-treated AD mice, we detected significant perfusion deficits in capillaries near (but not away from) Aβ plaques compared to WT mice (Fig. 5h,i). Moreover, cortical capillaries contained elevated numbers of blocked leukocytes (labeled for CD45), neutrophils (Ly6G) and erythrocytes (ter119) in AD mice compared to WT mice (Fig. 5h,j,k). Notably, in AD cerebellum (which largely lacks Aβ deposits), capillary perfusion and neutrophil block of capillaries were not affected (Extended Data Fig. 7i). The increase in leukocytes stuck in cortical capillaries in AD did not reflect higher blood leukocyte counts (Extended Data Fig. 7g and Supplementary Fig. 1). Furthermore, blocks with associated neutrophils occurred in close proximity to pericyte somata (Extended Data Fig. 7h), suggesting that blocks are driven by pericytes constricting capillaries. Indeed, we found that, in AD mice treated for 1.5 h with nimodipine in vivo, capillary perfusion was largely restored, and the number of stalled leukocytes and erythrocytes was reduced to below those of WT counts (Fig. 5i–k). Thus, nimodipine increases CBF in AD in several ways: by reversing the reduction of capillary diameter that occurs (thus also maintaining the blood viscosity lower^18^) and by reducing the subsequent block of capillaries by leukocytes^19^ and erythrocytes.

### Nimodipine improves CBF and reduces brain hypoxia in AD mice

**Fig. 6:**
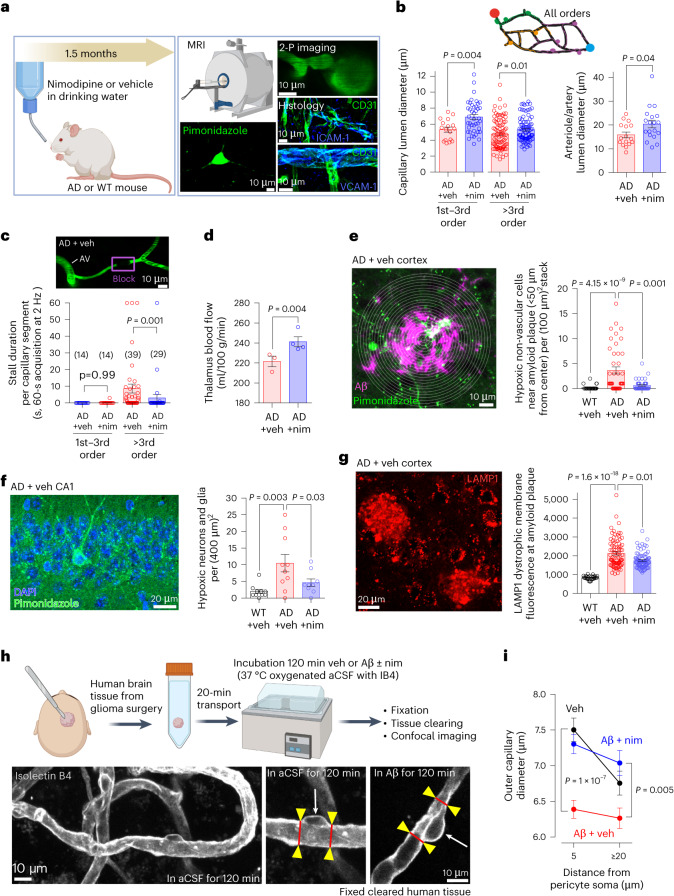
Nimodipine improves CBF and reduces brain hypoxia in AD mice and reduces Aβ-evoked pericyte contraction in human brain tissue. **a**, Schematic of long-term nim or vehicle treatment via the drinking water for 1.5 months starting from 2–3 months of age in WT and AD mice and use of mice for MRI, hypoxia assessment or immunohistochemistry (created with BioRender). **b**, Long-term nim increased the diameter of capillaries, arterioles and arteries in AD cortex. *P* values are from unpaired *t*-test for 1st–3rd order capillaries and Mann–Whitney tests for >3rd order and arterioles/arteries. **c**, Nim reduces stalling in >3rd order capillaries in AD mice (see also Supplementary Video 10). *P* values are from Mann–Whitney tests. **d**, CBF (from MRI) was increased in the thalamus of AD mice treated with nim (each point is one animal). *P* value is from unpaired *t*-test. **e**,**f**, AD increased and nim reduced in vivo labeling with pimonidazole (to identify hypoxia) in the cortex (**e**) and hippocampus (**f**) (*P* values are from Kruskal–Wallis test and one-way ANOVA, respectively). **g**, Nim reduces plaque-associated labeling with LAMP1 (*P* values are from Kruskal–Wallis test). **h**,**i**, Effect of nim on human pericytes. **h**, Top schematic: human tissue from neurosurgery was transported on ice-cold aCSF, incubated for 120 min in aCSF or Aβ with or without nim and fixed for tissue clearing and confocal imaging (created with BioRender). Bottom schematic: specimen images of fixed human cortical tissue incubated in aCSF (left image and middle zoom-in image) or 75 nM soluble Aβ oligomers^12^ (right image) when alive and labeled with isolectin B4 to identify pericytes (arrows) and measure capillary diameter (red lines at yellow arrowheads). **i**, Mean capillary diameter at approximtely 5 µm and ≥20 µm from the pericyte soma in human cortical tissue incubated in aCSF (*n* = 99 pericytes) or Aβ in the absence (*n* = 145) or presence (*n* = 120) of nim (*P* values are from one-way ANOVA for 5-µm distance and from Kruskal–Wallis test for ≥20-µm distance; additional *P* values are stated in the main text). *P* values are two-tailed. Error bars are s.e.m. 2-P, two-photon; nim, nimodipine; veh, vehicle. Source data

**Extended Data Fig. 8:**
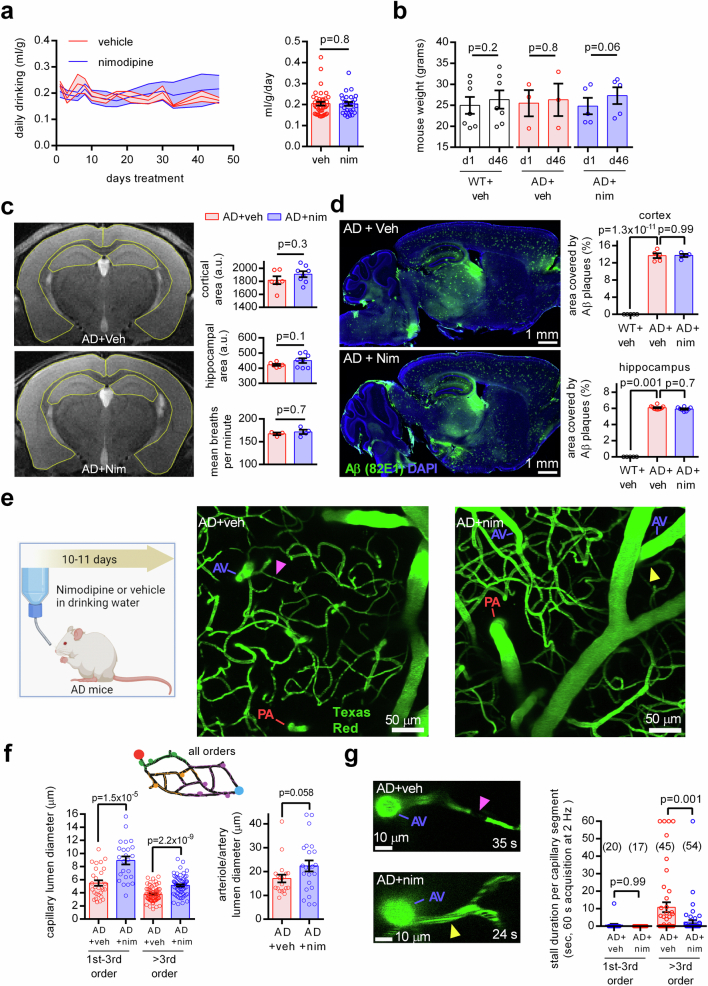
Drinking water intake, mouse weight, brain volume and plaque load are unchanged by long-term nimodipine treatment in AD mice. **a** Daily drinking volume per AD mouse weight of nimodipine or vehicle (DMSO) supplemented water over 1.5 months. **b** Nimodipine does not modulate mouse weight compared to vehicle treatment alone. **c** High resolution in vivo MRI scans of AD mice treated for 1.5 months with vehicle or nimodipine supplemented drinking water. Yellow lines outline hippocampal and cortical regions. Nimodipine treatment does not affect cortical area, hippocampal area or breathing rate during MRI in isoflurane-anesthetised AD mice in vivo. **d** Axioscan images of sagittal sections stained for Aβ (82E1) from AD mice treated for 1.5 months with vehicle or nimodipine. Aβ plaque load quantified in the cortex or hippocampus was not modulated by nimodipine treatment. **e-g** In vivo two photon-imaging in the barrel cortex of anesthesised AD mice 10-11 days after nimodipine or vehicle treatment (**e**) showed that nimodipine increases capillary (but not significantly arteriole/artery) diameters (**f**) and reduces capillary stalling in >3^rd^ order capillaries (**g**). P values in panels **a**, **c** and **g** are from Mann-Whitney tests, in panel **b** from paired t-tests, in panel **d** from a one-way ANOVA and a Kruskal^−^Wallis test for cortex and hippocampus, respectively, and in panel **f** from an unpaired t-test. P-values are 2-tailed. Error bars are s.e.m. Source data

Having established how nimodipine reverses the decrease of CBF in AD mice, to mimic a clinical scenario for early AD treatment (assuming earlier AD diagnosis) we treated AD mice with nimodipine or vehicle in the drinking water for 1.5 months (Fig. 6a and Extended Data Fig. 8a,b), starting at an age (2–3 months) marking the onset of synapse loss and Aβ plaque deposition but long preceding memory loss^25^. Using in vivo two-photon imaging, we found that nimodipine treatment significantly increased capillary diameters throughout the cortical vasculature (Fig. 6b) and largely reduced blood stalling^19^ in capillaries of >3rd branching order from arterioles (Fig. 6c and Supplementary Video 10). Stalls were largely absent in 1st–3rd order capillaries of vehicle-treated or nimodipine-treated mice (Figs. 5c and 6c). To test whether nimodipine also modulates CBF in subcortical regions (not accessible by two-photon microscopy, laser Doppler flowmetry or laser speckle imaging), we used arterial spin labeling (ASL) MRI to non-invasively measure CBF in the thalamus. This revealed that long-term nimodipine treatment significantly increased thalamic CBF in AD mice (Fig. 6d).

Measuring cerebral hypoxia with pimonidazole in vivo showed that AD increased the number of hypoxic cells in the cortex near Aβ plaques^12^ and in the hippocampus, and the numbers of hypoxic cells were greatly reduced after 1.5 months of nimodipine treatment (Fig. 6e,f). Nimodipine also significantly reduced the number of plaque-associated lysosomes (in microglia and axonal swellings) labeled^60^ for lysosomal-associated membrane protein 1 (LAMP1) (Fig. 6g). These nimodipine-evoked changes were unlikely to be biased by changes in brain atrophy because cortical and hippocampal areas were similar in vehicle-treated and nimodipine-treated mice (from MRI in vivo; Extended Data Fig. 8c).

**Extended Data Fig. 9:**
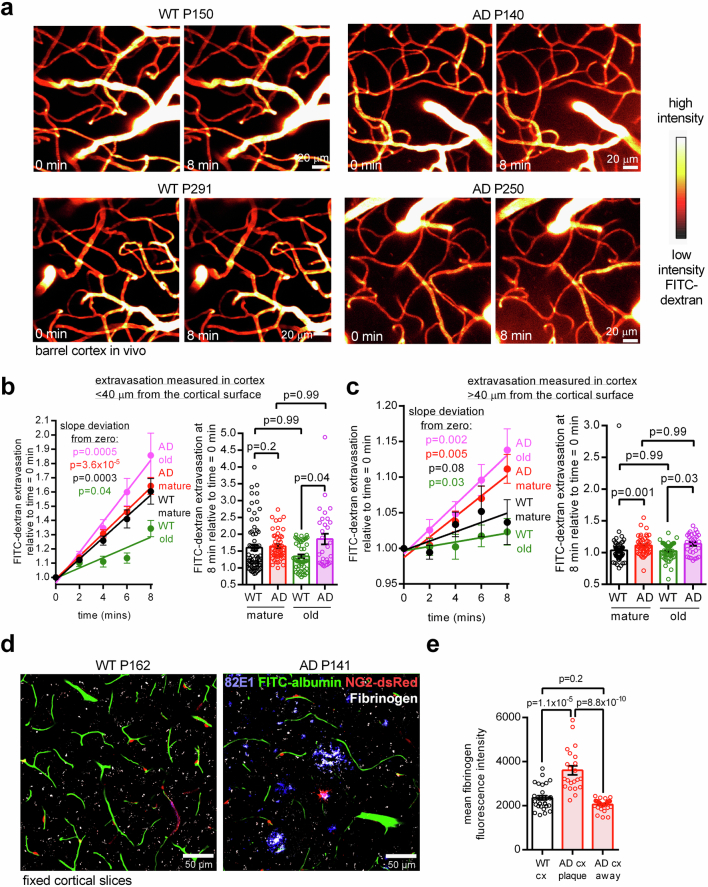
FITC-dextran extravasation and plaque-associated fibrinogen deposition are increased in AD mice. **a** Two-photon images (projections with all pixels summed) in barrel cortex at 0 min and 8 min corresponding to 2 min and 10 min after injection of 70 kDa FITC-dextran into the femoral vein of mature (upper panel) or old (lower panel) WT and AD mice in vivo. **b-c** FITC-dextran extravasation measured as the mean intensity increase in FITC-dextran signal in the extravascular space as a function of time from injection. FITC-dextran fluorescence was quantified from images as in (**a**) in the most superficial 40 μm of the cortex including the pia and part of layers I/II (**b**) and in the region from 40–126 μm deep in layers I-II (**c**). FITC-dextran extravasation is significantly increased in both mature and old AD mice at depths >40 μm, and is increased from pial and intraparenchymal vessels in the most superficial 40 μm of old but not mature AD mice. Points on bar chart are data from individual extravascular regions for which FITC-dextran was quantified. Mature mice were aged P120-180 (WT) and P125-156 (AD), and old mice were aged P208-361 (WT) and P211-364 (AD). **d** Fibrinogen and amyloid β (Aβ) plaque (82E1) staining in fixed cortical tissue sections of mature NG2-dsRed WT and AD mice that underwent cardiac perfusion with FITC-albumin in gelatin (see Methods). **e** Cortical fibrinogen deposits are elevated at plaques in cortex of AD mice. The fluorescence intensity of the fibrinogen channel was measured from multiple regions of interest in the extravascular space for each confocal stack and then averaged for each stack. Each point on bars is a different confocal stack. P values in panels **b**, **c** and **e** are from Kruskal-Wallis tests. P-values are 2-tailed. Error bars are s.e.m. Source data

Nimodipine treatment for 1.5 months did not modulate endothelial expression of ICAM-1 or VCAM-1 in capillaries, arterioles or venules (Extended Data Fig. 7a–f) nor did it modulate FITC-dextran extravasation in three vehicle-treated and four nimodipine-treated mature AD mice (*P* = 0.6 and *P* = 0.8, from Mann–Whitney tests, for extravasation measured in cortex at depths <40 µm and 40–126 µm from the cortical surface, respectively). FITC-dextran extravasation and fibrinogen deposition were enhanced in untreated AD mice compared to WT mice (Extended Data Fig. 9a–e). For FITC-dextran, extravasation was greatest in the 40-μm zone nearest the cortical surface (Extended Data Fig. 9b,c). AD significantly increased extravasation in mature and old mice at more than 40 μm below the cortical surface (Extended Data Fig. 9c), whereas, at depths less than 40 μm, AD increased extravasation for old but not mature mice (Extended Data Fig. 9b). In AD, fibrinogen deposition was greatest near amyloid plaques (Extended Data Fig. 9d,e). No differences in water consumption, body weight, breathing rate or Aβ plaque load were detected between vehicle and nimodipine groups after 1.5 months of treatment (Extended Data Fig. 8a–d). In a second cohort of AD mice treated for 10–11 d with vehicle or nimodipine in the drinking water, we detected a similar increase in the diameters of capillaries and a decrease in capillary stalling (Extended Data Fig. 8e–g), suggesting that nimodipine restores CBF early on in the 1.5-month treatment course and that this improvement persists for at least 1.5 months of nimodipine treatment (Fig. 6b).

**Extended Data Fig. 10:**
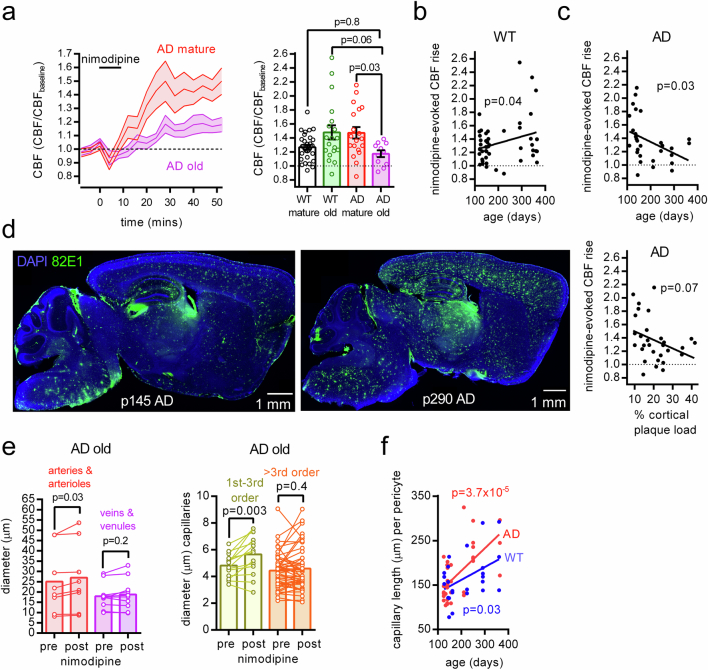
Ageing reduces the nimodipine-evoked increase in CBF in AD, but not in WT mice. **a** Time course of CBF in response to nimodipine in all mature and old WT and AD mice studied regardless of dsRed or NG2-Cre^ERT2^-GCaMP5g co-expression. Bar graph: Ageing reduces the nimodipine-evoked increase in CBF in AD, but not in WT mice (p = 0.1, Mann-Whitney test comparing mature to old WT mice, see also Fig. 1c, f). Mature mice were aged P110-P191 and old mice are aged P211-P364. **b** In WT mice, ageing increases nimodipine-evoked CBF rises. **c** In AD mice, nimodipine-evoked CBF rises decrease with age. **d** Axioscan images of fixed P145 and P290 AD sagittal sections labelled with DAPI and for Aβ using the 82E1 antibody. Nimodipine-evoked CBF rises negatively correlated with plaque load. **e** Left bar graph: nimodipine evokes a small dilation of arteries/arterioles but not veins/venules in old AD mice. Right bar graph: nimodipine increases 1^st^-3^rd^ order capillary diameter but does not alter >3^rd^ order capillary diameter at pericyte somata in old AD mice. **f** Pericyte loss during ageing in WT and AD mice. P values in panel **a** are from a Kruskal-Wallis test and in panel **e** from a paired t-test except for ‘>3^rd^ order’, which is from a Wilcoxon test. P values in b, c, d and f assess whether regression lines have a slope significantly different from zero. P-values are 2-tailed. Error bars are s.e.m. Source data

Because CBF declines in the aging AD brain^11^, we sought to investigate the effect of acute nimodipine treatment on CBF in aged WT and AD mice. Using laser Doppler flowmetry, we found that the nimodipine-evoked CBF increase was significantly attenuated (by 63%, *P* = 0.03) in old (>P200) AD mice compared to mature (P110–P200) AD mice (Extended Data Fig. 10a). Although, in WT, the nimodipine-evoked CBF rises correlated positively with aging, in AD nimodipine-evoked CBF, rises correlated negatively with aging and possibly cortical Aβ plaque load (Extended Data Fig. 10b–d). Although, in old AD mice, arteries, arterioles and 1st–3rd order capillaries dilated in response to nimodipine, the nimodipine-evoked dilation of >3rd order capillaries was largely absent within 1 h of treatment (Extended Data Fig. 10e). In late AD, this, together with (1) an accelerated loss of pericytes^61^ (Extended Data Fig. 10f), which will lead to BBB breakdown (partly by upregulation of leukocyte adhesion molecules^62,63^), (2) the downregulation of mural cell CaVs (Extended Data Fig. 3e) and (3) the loss of synapses and neurons^64^ implies that mural cell [Ca^2+^]_i_-lowering drugs may offer the greatest benefit for prophylaxis or early treatment of AD.

### Nimodipine reverses Aβ-evoked pericyte contraction in human cortical tissue

To test whether CaVs contribute to Aβ-evoked pericyte contraction in humans^12^, we incubated live human cortical tissue with artificial cerebrospinal fluid (aCSF) alone or with soluble Aβ oligomers in the absence or presence of nimodipine (Fig. 6h). In aCSF alone, the outer capillary diameter was significantly more dilated near to, compared to away from, pericyte somata (*P* = 0.0003, Mann–Whitney *t*-test; Fig. 6i) as reported previously^12,34^, possibly reflecting pericyte somatic release of factors that induce growth of the endothelial tube. Applying Aβ oligomers significantly constricted capillaries near pericyte somata (*P* = 10^−7^, one-way ANOVA with Tukey’s post hoc test) but evoked no significant diameter decrease away from pericyte somata (*P* = 0.08, Kruskal–Wallis test with Dunn’s post hoc test) (Fig. 6i). However, when Aβ oligomers were applied in the continuous presence of nimodipine, capillary diameters at pericyte somata were not significantly different from those of vehicle-treated capillaries (*P* = 0.5, Kruskal–Wallis test with Dunn’s post hoc test) (Fig. 6i). Thus, in live human brain tissue, Aβ-evoked pericyte contraction is driven by activation of L-type CaVs, presumably of the Ca_V_1.2 subtype expressed in human pericytes (Extended Data Fig. 3). Consequently, in humans with AD, nimodipine should increase capillary diameter and decrease hypoxia, as in mouse.

## Discussion

We targeted several mechanisms causing capillary constriction by pericytes in AD^12^: Ca^2+^ entry via L-type calcium channels using the BBB-permeable blocker nimodipine; amplification of [Ca^2+^]_i_ rises^14^ by the Ca^2+^-gated Cl^−^ channel TMEM16A using its blocker 10bm; and the contribution of ROS to pericyte contraction^12^ using antioxidants (NAC and GSK). These agents reduced pericyte-mediated capillary constriction (Figs. 2–4 and Extended Data Fig. 1) and increased CBF. Pericyte relaxation by nimodipine not only directly increased CBF by reducing vascular resistance; it also reduced the number of leukocytes and erythrocytes that became trapped near pericytes and stalled capillary blood flow (Fig. 5i–k). Consequently, hypoxia in the AD brain was removed by nimodipine (Fig. 6e,f). Thus, targeting CaVs, TMEM16A or ROS might prove useful for therapy development to maintain CBF and improve neuronal function during AD.

Although most mechanistic experiments in this study were on anesthetized mice, we also found that nimodipine dilated capillaries and arterioles/arteries of awake unanesthetized mice (Extended Data Fig. 1h,i) and that giving nimodipine for 1.5 months in the drinking water to unanesthetized AD mice evoked pericyte relaxation, increased blood flow and reduced capillary stalling, tissue hypoxia and dystrophic membrane labeling (Fig. 6a–g). In human tissue, nimodipine reversed Aβ-evoked, pericyte-mediated capillary constriction (Fig. 6h,i), indicating that CBF could be increased in patients developing AD using BBB-permeable CaV blockers.

Most of the more than 400 putative therapies targeting AD have not prevented cognitive decline, probably because they are given after irreversible brain damage has occurred^1^. This suggests that successful future therapies for AD will involve either prophylaxis (just as BP-lowering drugs are given to reduce the risk of stroke^65^) or, if specific biomarkers are developed, treating the earliest stages of the disease. Targeting the CBF decrease that occurs early in AD and reaches 45% in affected areas^3,8,9,12^, sufficient to cause cognitive changes and neuronal cell damage^4–6^ (see Main), is attractive, because CBF can be measured relatively non-invasively using dynamic susceptibility contrast (DSC) MRI^7^ (and possibly, in the future, using ASL MRI techniques).

In human patients and in mice, capillary pericytes constrict capillaries early in AD^12^. In mice, this occurred with no diameter change in arterioles or venules, implying that the CBF reduction early in AD is generated by pericytes. Pericyte-mediated capillary constriction reduces CBF because of the diameter decrease (via Poiseuille’s law), the smaller diameter increasing blood viscosity^18^ and by inducing capillary block by blood cells^19^. Capillary block can also result from upregulation of endothelial adhesion molecules induced by a transgenically induced loss of pericytes from capillaries^63^, which may mimic the pericyte loss occurring late in AD^61^ (Extended Data Fig. 10f). In AD mice, neutrophils, monocytes and erythrocytes all contributed to producing capillary blocks near pericytes (Fig. 5), which may, in the long term, lead to capillary loss^66^.

We focused on CaVs because of clinically available drugs targeting these channels that might be repurposed for prophylaxis or early treatment of AD. Some epidemiological analyses^67–71^ found less cognitive decline in patients treated with CaV blockers, although many such blockers have poor BBB permeability, and they may act by lowering BP (autoregulation mechanisms oppose this but may weaken in AD^72^). The nimodipine-evoked fall of mural cell [Ca^2+^]_i_ and in vivo vessel diameter increases showed directly that nimodipine relaxes both pericytes (evoking dilation near pericyte somata) and SMCs around arterioles, which will both increase CBF. Presumably, nimodipine suppresses Ca^2+^ entry into these cells, thus evoking dilation (as when Ca_V_1.2 is deleted in mural cells^73^). We considered whether nimodipine might also act on neurons to reduce production of ROS that trigger contraction^12^, but this seems unlikely because we saw no increase in ROS production in cells other than microglia or perivascular macrophages (Fig. 4c,d and Extended Data 5b,k). Nimodipine did not affect the area occupied by Aβ plaques (Extended Data Fig. 8d), suggesting that it does not alter Aβ production, and an action on NO release is unlikely to explain our results: if nimodipine acted on endothelial or neuronal L-type Ca^2+^ channels, it would lower [Ca^2+^]_i_, decrease NOS activation and decrease NO release, thus promoting constriction rather than dilation. Furthermore, considering the nimodipine concentration reached in the central nervous system (CNS), its speed of action and its half-maximal inhibitory concentration (IC_50_) for calcium channel inhibition or acting via other potential mechanisms indicates that only calcium channel inhibition is likely to contribute to increasing CBF (see Supplementary Information).

The APP^NL–G–F^ mice that we study provide a good model of the decreased CBF and elevated Aβ production that occur in AD, but show only minor behavioral changes^74–76^, especially when 4 months of age, which we chose aiming to prevent the earliest stages of AD. This highlights a need to determine how longer-term nimodipine treatment affects cognitive function.

The concept that preventing the pericyte-mediated fall of CBF that occurs in early AD^12^ could significantly ameliorate cognitive decline is supported by data showing that an antibody preventing neutrophil stalling in capillaries, which increases CBF, improved cognition into late AD in mice^77^. However, in a phase 3 clinical trial, a nimodipine-related drug, nilvadipine, which also increases CBF, did not reduce cognitive decline in human patients with mild to moderate AD^78^, perhaps because the drug was started only 4.3 years after symptom onset. In addition, although reduced CBF is an early result of Aβ generation in AD^12^, Aβ also has other effects^79^, including inhibiting glutamate uptake^80,81^ (which alters neural excitability) and promoting tau hyperphosphorylation (which disrupts synaptic function)^82,83^. Thus, therapies targeting Aβ oligomer production and tau phosphorylation may synergize with therapies aimed at maintaining CBF, such as nimodipine.

## Methods

### Animals

Mice (either sex) in individually ventilated cages (12-h light/dark cycle, 21 ± 1 °C, 45–65% humidity) were fed normal chow and tap water ad libitum. Cages contained Aspen chip and Sizzle-Nest bedding and an environmental enrichment tunnel. Crossing NG2-dsRed mice^21^ with APP^NL–G–F^ mice (termed AD mice here)^25^ gave APP^NL–G–F^NG2-dsRed mice, which was chosen as an AD model that shows increased Aβ production and aggregation (via Swedish, Arctic and Iberian mutations of APP), with mutated APP being knocked-in at its endogenous locus to avoid overexpression artifacts^25^. Although they lack tau tangles, phosphorylated tau is elevated in dystrophic neurites around plaques. They model the decreased CBF and elevated Aβ production occurring in AD but show only minor behavioral changes^74,75^. ADxIba1-eGFP or ADxCX3CR1-GFP mice were obtained by crossing AD mice with Iba1-eGFP mice^84^ or heterozygous CX3CR1-GFP mice^85^. ADxNG2-Cre^ERT2^-GCaMP5G mice were generated by crossing AD mice with tamoxifen-inducible NG2-Cre^ERT2^ knock-in mice^86^ and floxed GCaMP5G-IRES-tdTomato mice^87^. Tamoxifen in corn oil was given (100 mg kg^−1^) by oral gavage daily for 4 days to adult >P21 mice. Mice had a C57BL/6J background. Animals for brain slicing were euthanized by cervical dislocation or cardiac perfusion under terminal anesthesia. Animal breeding, experimental procedures and euthanization methods were in accordance with UK Home Office regulations (Guidance on the Operation of Animals, Scientific Procedures Act, 1986) and the advice of the UCL Animal Welfare Ethical Review Board or overseen by the Boston Children’s Hospital Institutional Animal Care and Use Committee following National Institutes of Health guidelines.

### Human tissue

Live human cortical tissue was from four males (aged 17, 28, 54 and 65 years) and one female (53 years) undergoing neurosurgical glioma resection at the National Hospital for Neurology and Neurosurgery, Queen Square, London, and informed consent was obtained (patients were not financially compensated). Cortical tissue above the glioma was removed (to gain access to the glioma) and transported to the laboratory in ice-cold HEPES-based aCSF containing (in mM): 140 NaCl, 10 HEPES, 2.5 KCl, 1 NaH_2_PO_4_, 10 glucose, 2 CaCl_2_, 1 MgCl_2_ (pH 7.4). After 30-min pre-incubation in oxygenated HEPES-based aCSF at 37 °C with or without nimodipine (3 μM), tissue was maintained in aCSF or transferred to aCSF containing Aβ (75 nM) with or without nimodipine for 120 min at 37 °C, supplemented with 10 μg ml^−1^ Alexa Fluor 647–conjugated IB4 (Invitrogen, I32450), which binds to microglia and capillary basement membranes^88^, allowing identification of microglia and pericytes. Pericytes embedded in basement membrane were identifed by their bump-on-a-log morphology (Fig. 6h) or position at capillary branch points. Tissue was fixed in 4% paraformaldehyde (PFA) at room temperature (1 h) shaking, cleared overnight in CUBIC reagent 1 solution (prepared (ref. ^89^) in distilled water at room temperature containing 25% w/v N,N,N′,N′-tetrakis(2-hydroxypropyl)ethylenediamine, 15% w/v Triton X-100 and 25% w/v urea), mounted in CUBIC reagent 1 and imaged using a Zeiss LSM 710 microscope. Diameters were measured on either side of pericyte somata (<5 µm from the center) and ≥20 µm away from the somata center by drawing lines across the IB4-labeled capillary in FIJI (ImageJ) and computing the full width at half maximum (FWHM) of a Gaussian fit to the fluorescence intensity profile of the line (see ‘Code availability’). All work had ethical approval from the National Health Service (REC number 15/NW/0568; IRAS project ID 180727).

### In vivo two-photon imaging and laser Doppler flowmetry

Craniotomies for acute in vivo imaging under terminal anesthesia were performed^14^. Mice were anesthetized using urethane (1.55 g kg^−1^ in two doses 15 min apart; Sigma-Aldrich, 94300), and a heating pad maintained body temperature at 36–37 °C. Eyes were protected from drying with polyacrylic acid eye drops (Dr. Winzer Pharma). The trachea was cannulated, and mice were mechanically ventilated with medical air supplemented with oxygen using a MiniVent (model 845). For slow nimodipine infusions (to a total of 220 μg kg^−1^; Sigma-Aldrich, N149; see Supplementary Material for brain concentration) or vehicle (4.78 mM PEG-400 (Sigma-Aldrich, 202398) dissolved in 15% (2-hydroxypropyl)-β-cyclodextrin (Sigma-Aldrich, H107) in PBS), the right femoral vein was cannulated using a catheter filled with sterile saline supplemented with heparin (20 IU) connected to a pump. The skull was exposed, thinned using a drill and dried using compressed air. A custom-made headplate was centered over the right barrel cortex, 3 mm laterally from the midline and immediately caudal to the coronal suture. The headplate was attached using superglue gel, and mice were headfixed to a custom-built stage. The skull was thinned over the left frontal cortex to attach one or two laser Doppler flowmetry probes approximately 5 mm apart (statistical tests were performed on 1–2 points per animal from 1–2 Doppler probes). CBF was measured with an OxyFlo Pro laser Doppler system (collected using MATLAB 2015b). A craniotomy (~2-mm diameter) was performed and the dura removed. The brain was sealed with 2% agarose in HEPES-buffered aCSF (33–37 °C) and covered by a glass coverslip or left exposed for H_2_O_2_ and GSK2795039 experiments (for which 1–2 laser Doppler probes were placed onto the exposed barrel cortex to measure CBF). To assess internal vessel diameters or BBB leakage, 70-kDa FITC-dextran (50 µl of 50 mg ml^−1^ in saline; Sigma-Aldrich, 46945) was given by slow retro-orbital injection or via the femoral vein. For leukocyte imaging, the neutrophil marker Alexa Fluor 488 anti-mouse Ly6G was given at a low dose (0.1 mg kg^−1^ i.v. in PBS; BioLegend, 127626) that does not induce neutropenia^19^, and sometimes the monocyte marker Brilliant Violet 421 anti-mouse F4/80 (0.1 mg kg^−1^ i.v. in PBS; BioLegend, 123137) was co-injected.

For awake in vivo imaging, the anti-inflammatory dexamethasone (2 mg kg^−1^) and the analgesic Ethiqa XR (3.25 mg kg^−1^) were given subcutaneously, and a craniotomy (~3.5-mm diameter) was performed over the right cortex using aseptic techniques under isoflurane anaesthesia (3% induction, 1.5% maintenance) with body temperature maintained at 36–37 °C. A stack of two 3-mm coverslips on top of one 5-mm coverslip (glued together using Norland Optical Adhesive 81) was placed onto the intact dura (with the 3-mm coverslip contacting the dura), and the stack was secured to the skull at the edges of the 5-mm coverslip with Vetbond (3M, 1469SB). Using C&B Metabond (Parkell), a custom-made titanium headplate was attached to the skull, and the cranial window was sealed. Mice were injected with 500 μl of saline (subcutaneously) and recovered in a heated cage until regaining motor function. Mice were allowed to recover from surgery for at least 2 weeks and then habituated to head fixation, running on a custom-built running wheel and awake imaging in the dark for ≥1 week before experiments. FITC-dextran (50 µl of 50 mg ml^−1^ in saline), nimodipine or vehicle were administered retro-orbitally under brief isoflurane anaesthesia (3%), and mice were allowed to recover from anesthesia before awake imaging.

Image z-stacks (0.65–2-μm step size, 138–420-nm pixel size, 1.58–3.14-μs pixel dwell time) were acquired in cortical layers I–III using a Newport / Spectra-Physics Ti:Sapphire Mai Tai laser pulsing at 80 MHz and a Zeiss LSM 710 microscope with a ×20 water immersion objective (numerical aperture (NA) 1.0) or an Olympus FVMPE-RS microscope with a ×25 water immersion objective (NA 1.0). Data were collected using ZEISS ZEN 2011 or Olympus FV31S-SW FluoView. Mean depth imaged below the cortical surface was 159 μm for WT and 169 μm for AD in Figs. 1, 2, 3a–c and 5a, 195 μm for WT in Fig. 3d–h, 76 μm for WT in Fig. 4a,b (see also Fig. 4a, inset), 204 μm for AD in Fig. 4g, 193 μm for AD in Fig. 6b,c, 314 μm for WT in Extended Data Fig. 1h,i, 161 μm for AD in Extended Data Fig. 2d, 221 μm for AD in Extended Data Fig. 8e–g and 178 μm for AD in Extended Data Fig. 10e. Image z-stacks were acquired every 1–2 min, images in a single plane at 1–2 Hz or at a slower rate down to 1 per 20 s and images for awake imaging before and approximately 20 min after nimodipine or vehicle administration. Fluorescence was evoked using a wavelength of 1,000 nm for dsRed and FITC-dextran, 940 nm for GCaMP5G (at which fluorescence is Ca^2+^sensitive), tdTomato and Texas Red or 920 nm for Iba1-eGFP. When GCaMP5G was excited at 800 nm (at which its fluorescence lacks Ca^2+^sensitivity), no fluorescence transients (and no photobleaching) were observed in AD mouse pericytes (Extended Data Fig. 2e–g; see also Fig. 7b in ref. ^14^ (sham aCSF) for lack of photobleaching of the GCaMP5g signal). Mean laser power in the focal plane was less than 25 mW, except when inducing laser injury (in a single plane within a ~7 µm^2^ focal point ~15–20 µm away from targeted vessel segments) by tuning the laser to 800 nm and setting the power to 220 mW and pixel dwell time to 177.3 µs. When needed, CBF recordings were obtained between imaging stacks to avoid the two-photon laser affecting the Doppler signal.

FITC-dextran extravasation indicated BBB permeability^90^ and was assessed by acquiring z-stacks every 1–2 min for 10 min, starting less than 2 min after FITC-dextran injection. After injection, the FITC-dextran fluorescence in the blood vessels was essentially constant: the intensity after the 8-min period in Extended Data Fig. 9a–c was not significantly different from initially (decreased by 4%, *P* = 0.9 from Mann–Whitney test). After stacks were z-projected summing pixel intensities at all depths, and co-registered using the StackReg plugin in FIJI, the FITC-dextran fluorescence increase in the extravascular milieu (10 regions per mouse) relative to the first acquired frame was quantified. Pericyte GCaMP5G fluorescence was quantified in the soma and processed in FIJI, normalizing each intensity value to the baseline average. [Ca^2+^]_i_ transients were detected if their amplitude exceeded 3× the standard deviation of the baseline average. Microglial surveillance was analyzed^91^ (see ‘Code availability’).

Outer and inner vessel diameters were measured as, respectively, the FWHM (for tdTomato-labeled pericyte processes) or full width at quarter maximum (FWQM) (for dye-labeled solution in the vessels) obtained using a FIJI macro that fits a Gaussian curve to a plot of the fluorescence intensity versus distance across the vessel (see ‘Code availability’). For some vessels, a Gaussian curve fitted poorly due to the accumulation of dye at the edges of the vessel wall. Diameters for these vessels were measured manually in FIJI. Capillary diameters were typically measured as a function of distance from pericyte somata except when pericytes were not transgenically labeled, as for Fig. 6b and Extended Data Figs. 1h,i and 8e,f, for which diameters were measured from a single randomly placed line on each vessel segment. In a subset of 1st–3rd order capillaries from Fig. 6b, we measured the diameters at 2–5 locations on each vessel segment and averaged them to give one mean value per segment. Comparing these mean values between 14 AD+vehicle measurements and 18 AD+nimodipine measurements again showed a significant difference (5.4 µm versus 6.8 µm, respectively, *P* = 0.003, unpaired *t*-test). Motility was assessed by normalizing the microglial surveillance index to the microglial area for each frame (measured from the maximum intensity projection of the binarized microglia cell in FIJI).

### Blood pressure

Mice were anesthetized using urethane (1.3 g kg^−1^) and mechanically ventilated (medical air supplemented with oxygen), and laser Doppler probes were attached to the thinned skull over the left and right barrel cortices to record CBF. Mice were placed in a thermal cage set to approximately 40 °C. Using a tail cuff attached to a CODA non-invasive blood pressure monitor (sCODA software 4.1) and allowing mice to acclimatize for 10 min, BP and CBF were measured before and after intravenous nimodipine administration (220 μg kg^−1^ total, over ~5.5 min, 60 mg ml^−1^ solution).

### Laser speckle imaging

After dura removal and placement of a glass cranial window over the right barrel cortex (as for in vivo imaging), CBF imaging was performed using a RFLSI III Laser Speckle Imaging System (version 1.0 software). CBF was measured in three capillary regions per mouse between pial vessels (exposure time, 5 ms). Laser speckle provides qualitative measures of CBF when comparing animals of different genotypes.

### Long-term nimodipine treatment in the drinking water

Drinking water in light-protected bottles was supplemented with nimodipine (40 µM; Bio-Techne, 0600) or vehicle (DMSO) for group-housed mice for 10–11 d or 1.5 months before MRI, two-photon imaging and/or in vivo hypoxia labeling. Volume of water consumed and weight per mouse were recorded every 1–5 d to estimate nimodipine intake per day (~3.3 mg kg^−1^ d^−1^) by dividing water volume consumed per cage by total weight of mice per cage.

### MRI measurement of CBF

Anesthesia was maintained using 1.5% isoflurane in an 80%/20% mixture of air/oxygen (free breathing). Mouse temperature was monitored rectally, and respiration was recorded using a pressure sensor. Images were acquired on a horizontal-bore 9.4T preclinical system (BioSpec 94/20 USR, Bruker) using a 440-mT/m gradient set with outer and inner diameters of 205 mm and 116 mm, (BioSpec B-GA 12S2), an 86-mm volume transit RF coil and a four-channel receiver array coil for mouse brain (Bruker, using ParaVision 6.1 software). A single-slice flow-sensitive alternating inversion recovery ASL MRI protocol was implemented using an interleaved slice-selective inversion pulse thickness of 4.5 mm and a global non-selective pulse. Imaging parameters were as follows: inflow times (TI) 100, 200, 500, 1,000, 1,500, 2,000, 3,000 and 4,000 ms; echo time 12 ms; pulse repetition time (TR) 10,000 ms; data matrix 64 × 64; field of view 20 × 20 mm; slice thickness 1.5 mm; six repetitions. To quantify CBF, T1 and equilibrium magnetization were extracted from the fit of the control signal as a function of TI, and the ASL signal was then fit to a two-compartment model^92^ (fitting arterial transit time and temporal duration of the tagged bolus).

### In vivo hypoxic tissue labeling

Hypoxia assessment used a pimonidazole HCl Hypoxyprobe Plus Kit (HP2-100, Hypoxyprobe, Inc.). Awake mice were interperitoneally injected with 60 mg kg^−1^ pimonidazole HCl. Pimonidazole rapidly crosses the BBB and binds thiols in hypoxic cells with a plasma half-life of approximately 30 min (ref. ^93^) in normal tissue and 15 min in hypoxic tissue. To minimize the death-induced binding of reduced thiols to hypoxyprobe, mice were anesthetized and cardiac perfused (9 ml min^−1^) with ice-cold PBS followed by ice-cold PFA at 6–7 h after injection.

### Staining whole blood for flow cytometry

Mouse tail veins were nicked using a 29-gauge needle, and whole blood was collected into PBS containing 5 mM EDTA. After removal of supernatant (centrifugation 400*g* for 5 min at 4 °C), cells were incubated with ACK lysis buffer (150 mM NH_4_Cl, 1 mM KHCO_3_, 0.1 mM EDTA) for 5 min at room temperature under gentle agitation, and MACS buffer was added (2% serum, 1 mM EDTA, PBS). Supernatant was removed (centrifugation 400*g* for 5 min), and cells were incubated with Pacific Blue anti-mouse CD45 (2.5 µg ml^−1^; BioLegend, 103125) or Alexa Fluor 647 anti-mouse Ly6G (2.5 µg ml^−1^; BioLegend, 127610) antibodies for 30 min at 4 °C in darkness. Supernatant was removed; cells were washed twice in MACS buffer by removing supernatant; and 123count eBeads counting beads (Thermo Fisher Scientific, 01-1234-42) were added to count live cells using a BD LSRFortessa Cell Analyzer. Data were collected with Diva 9.0.1 and analyzed with FlowJo 10 software.

### Acute brain slice imaging

Brain slices 300 μm thick were prepared using a Leica VT1200S vibratome in ice-cold, oxygenated (95% O_2_/5% CO_2_) slicing solution^14^ containing (in mM): 93 *N*-methyl-d-glucamine chloride (NMDG-Cl), 2.5 KCl, 30 NaHCO_3_, 10 MgCl_2_, 1.2 NaH_2_PO_4_, 25 glucose, 0.5 CaCl_2_, 20 HEPES, 5 Na-ascorbate, 3 Na pyruvate and 1 kynurenic acid. For DHE experiments, slices were incubated in this solution with 10 μg ml^−1^ Alexa Fluor 488–conjugated IB4 (Invitrogen, I21411) for 30 min at room temperature, washed briefly in slicing solution at room temperature and placed underneath a harp on a coverslip for imaging using an LSM 700 microscope. After acquiring one baseline stack of IB4-labeled microglia and 555-excited autofluorescence (320 × 320 × 40 µm, 2-µm z-step, 1.58-µs pixel dwell, 0.3-µm pixel size), slicing solution was replaced with the same solution containing 8 µM DHE (Cayman Chemical, 12013), and another stack was acquired. To measure ROS in Iba1-expressing microglia and NG2-expressing microglia of ADxIba1-eGFPxNG2-dsRed mice, slices were incubated with or without 50 µM NAC for 30 min, and image stacks were acquired before and after adding 5 µM CellROX Deep Red (Invitrogen, C10422) in the absence or presence of NAC. CellROX penetrated poorly into the slice, so all imaging was performed 40 µm below the slice surface (as for imaging DHE using the same settings). Stacks were maximum intensity projected in FIJI, and the DHE or CellROX signal in microglial somata was normalized to the autofluorescence (pre-DHE or pre-CellROX) signal. For calcium imaging, slices were incubated at 37 °C in slicing solution (20 min) and then transferred into solution at room temperature in which NMDG-Cl, MgCl_2_, CaCl_2_ and Na-ascorbate were replaced by (in mM) 92 NaCl, 1 MgCl_2_, 2 CaCl_2_ and 1 Na-ascorbate. Two-photon Ca^2+^ imaging in slices from NG2-Cre^ERT2^-GCaMP5G mice employed a Zeiss LSM 780 microscope with the two-photon laser (Ti:Sapphire Mai Tai DeepSee, Spectra-Physics) tuned to 940 nm. Slices were perfused (5 ml min^−1^) with aCSF (~34 °C) containing (in mM) 124 NaCl, 2.5 KCl, 26 NaHCO_3_, 1 MgCl_2_, 1 NaH_2_PO_4_, 10 glucose, 1 ascorbate and 2 CaCl_2_. The aCSF was gassed with 20% O_2_/5% CO_2_/75% N_2_. Image stacks were maximum intensity z-projected, co-registered and analyzed in FIJI. Experiments occurred less than 4 h after euthanizing mice.

### Immunohistochemistry for confocal and Axioscan imaging

Anesthetized mice (1.55 g kg^–1^ urethane) were cardiac perfused with ice-cold PBS followed by ice-cold PFA or with 20 ml of warm (34–37 °C) PBS with heparin (20 IU), followed by 20 ml of warm 0.25% (w/v) FITC-albumin (Sigma-Aldrich, A9771) in 5% (w/v) gelatin from porcine skin (Sigma-Aldrich, G1890) in PBS. FITC-perfused mice were placed head down into ice for 30 min. Brains were extracted and drop fixed in 4% PFA overnight, and 100-μm-thick slices were cut in PBS using a Leica VT1200S vibratome. After permeabilizing and blocking slices overnight at 4 °C in blocking buffer (10% (v/v) horse serum, 0.3% (v/v) Triton X-100, 1.5% (w/v) glycine and 1% (w/v) BSA in PBS), slices were incubated with primary antibody (82E1, 1:500, IBL 10323; P2Y_12_R, 1:200, AnaSpec AS-55043A; CD31, 1:100, R&D Systems AF3628; fibrinogen, 1:200, US Biological F4203-02F; CD206, 1:100, R&D Systems AF2535) or primary conjugated antibody (from BioLegend, 2.5 µg ml^−1^: ICAM-1, 116114; VCAM-1, 105712; Ly6G, 127610; CD45, 103125; and ter119, 116211; from Santa Cruz Biotechnology, 4 µg ml^−1^: gp91-phox (54.1) AF647, sc130543) in blocking buffer overnight at 4 °C. The same procedure was followed for LAMP1 (BioLegend, 121602, 1:200) staining except without permeabilization and using 20% donkey serum as the blocking buffer (to restrict labeling to dystrophic membrane of damaged or dead cells as well as plaque-associated microglia^60^). Slices were washed 4 × 10 min in PBS, incubated (where required) with the secondary antibody (1:500, from Thermo Fisher Scientific: anti-mouse Alexa Fluor 405, A-31553; anti-mouse Alexa Fluor 488, A-21202; anti-mouse Alexa Fluor 633, A-21050; anti-rat Alexa Fluor 633, A-21094; anti-rabbit Alexa Fluor 488, A21206; anti-sheep Alexa Fluor 633, A-21100; anti-goat Alexa Fluor 488, A-11055; anti-goat Alexa Fluor Plus 647, A32849) in blocking buffer overnight at 4 °C and washed again 4× before mounting. Some slices were co-incubated with 10 μg ml^−1^ Alexa Fluor 647–conjugated IB4. Confocal images acquired using an LSM 700 or an LSM 880 microscope (and ZEN 2011 or ZEN 2.3 SP1 FP3 (Black) software) were analyzed as follows. Perfused vessel segment lengths were quantified by tracing all FITC-albumin-filled vessels in three dimensions using the Simple Neurite Tracer plugin in ImageJ. These traces were overlayed with IB4 and NG2 channels to obtain the total vessel length including non-perfused vessel segments that are not filled with FITC-albumin. The length of FITC-albumin-filled vessels was expressed as a percentage of total vessel length. Capillary pericyte coverage was quantified as fluorescence intensity of tdTomato-labeled pericyte processes as a function of distance from the center of the pericyte soma (in z-projected images). Fluorescence was measured over a one-pixel-wide line in 1-µm increments up to 15 µm away from pericyte somata in the middle of the capillary lumen. Distance of the pericyte soma surface to the microglia soma surface was measured in three dimensions using IMARIS. NG2-expressing microglia were counted using the Cell Counter plugin in FIJI. ICAM-1 or VCAM-1 co-localization with CD31 was calculated by binarizing the CD31 channel, creating a mask and multiplying it by the raw ICAM-1 or VCAM-1 channel in FIJI. LAMP1 fluorescence was measured at plaques in a single plane using FIJI. Plaque area was calculated by dividing binarized plaque signal area by total area in images acquired with a Zeiss Axioscan Z1 fluorescence multi-slide scanner with a Plan-Apochromat ×10/0.45 M27 objective.

### Reagent preparation

The TMEM16A blocker was made as follows. First, 2-cyano-*N*-(*o*-tolyl)acetamide was prepared using cyanoacetic acid and *o*-toluidine (General Procedure 1 (ref. ^20^)) and converted to 2-cyano-2-cycloheptylidene-*N*-(*o*-tolyl)acetamide using cycloheptanone (General Procedure 2 (ref. ^20^)). This was converted to 2-amino-*N*-(*o*-tolyl)-5,6,7,8-tetrahydro-4*H*-cyclohepta[b]thiophene-3-carboxamide (General Procedure 2 (ref. ^20^)). Finally, this and 2-bromo-2,2-difluoroacetyl chloride were used to make 2-[(2-bromo-2,2-difluoro-acetyl)amino]-*N*-(*o*-tolyl)-5,6,7,8-tetrahydro-4*H*-cyclohepta[b]thiophene-3-carboxamide (General Procedure 3 (ref. ^20^)).

### Statistics and reproducibility

Data were analyzed using MATLAB (2011b, 2018b and 2019b), IMARIS (version 9.1) or FIJI (ImageJ 2.1.0); statistical tests were performed in R (version 3.4.2 or 3.6.3) and Prism 6 or 10 (GraphPad Software). Data show mean ± s.e.m. Drug treatments were randomly assigned. Investigators were blinded to group allocation during analysis and sometimes during data collection (MRI and immunohistochemistry), unless blinding was impossible due to autofluorescence of Aβ plaques in AD mice. Data with unstable CBF or drift in vessel diameter or pericyte [Ca^2+^]_i_ before drug application were excluded. Mice with unsuccessful cardiac perfusions or poor cranial window preparations (giving unstable imaging) were excluded. No predetermination of sample size was done, but sample sizes are similar to those used previously^12,14^. Data normality was assessed using D’Agostino–Pearson omnibus tests. For non-normally distributed data, a non-parametric statistical analysis was performed using Mann–Whitney *U*-tests (comparing two groups, unpaired), Kolmogorov–Smirnov tests (comparing cumulative distributions, unpaired), Wilcoxon tests with continuity correction (comparing two groups, paired) or Kruskal–Wallis tests with Dunn’s post hoc test (comparing more than two groups). For normally distributed data, a parametric test was used. *P* values were from homoscedastic (equal variance) or heteroscedastic (unequal variance, with Welch’s correction) Student’s *t*-tests (comparing two groups) or one-way ANOVAs with a Tukey, Dunnett or Holm–Sidak post hoc test (comparing more than two groups) as appropriate. These procedures included correction for multiple comparisons within each figure panel. To test whether capillary diameter changes with distance from pericyte soma, we assessed whether the slope of the linear regression fit significantly deviated from zero. *P* values less than 0.05 were considered significant, and all tests were two-tailed. *n* numbers for all figure panels are given in Supplementary Table 1. All representative images shown are typical of three or more animals.

### Reporting summary

Further information on research design is available in the Nature Portfolio Reporting Summary linked to this article.

## Online content

Any methods, additional references, Nature Portfolio reporting summaries, source data, extended data, supplementary information, acknowledgements, peer review information; details of author contributions and competing interests; and statements of data and code availability are available at 10.1038/s41593-024-01753-w.

## Supplementary information

Supplementary information Concentration of nimodipine used and mechanism of action. Supplementary References, Supplementary Fig. 1 and list of supplementary videos.

Reporting Summary

Supplementary Table 1 Number of male and female mice used for each figure panel

Supplementary Video 1 Pericytes of the 1st–3rd capillary branching order and >3rd capillary branching order with circumferential processes near their somata labeled using tdTomato (re-colored green) in the cerebral cortex of WT NG2-Cre^ERT2^-GCaMP5G mice in vivo and rendered in IMARIS (see also Extended Data Fig. 1a).

Supplementary Video 2 Nimodipine reduces [Ca^2+^]_i_ in processes and somata of 1st branch order pericyte and dilates capillary in the barrel cortex of an AD NG2-Cre^ERT2^-GCaMP5G mouse (see also Fig. 2f).

Supplementary Video 3 In vivo two-photon imaging of pericytes with excitation at 940 nm or 800 nm in the barrel cortex of an AD NG2-Cre^ERT2^-GCaMP5G mouse (see also Extended Data Fig. 2e–g). Repeated Ca^2+^ transients seen in the image excited at 940 nm are absent in the image excited at 800 nm.

Supplementary Video 4 Laser-evoked injury raises [Ca^2+^]_i_ and contracts >3rd branch order pericytes near their somata in the barrel cortex of WT NG2-Cre^ERT2^-GCaMP5G mice in vivo (see also Fig. 3d–h).

Supplementary Video 5 Laser-evoked injury does not raise [Ca^2+^]_i_ in PA SMCs and 2nd branch order capillary pericytes in the barrel cortex of a WT NG2-Cre^ERT2^-GCaMP5G mouse in vivo (see also Extended Data Fig. 4b–d).

Supplementary Video 6 In vivo two-photon imaging of capillary blocks near pericytes labeled with NG2-dsRed in the barrel cortex of an AD mouse with FITC-dextran in the blood (see also Fig. 5a).

Supplementary Video 7 In vivo two-photon imaging of Ly6G-labeled neutrophils or Iba1-eGFP-expressing monocytes in the vessel lumen (labeled with Texas Red in the blood) in the barrel cortex of an AD mouse (see also Fig. 5d).

Supplementary Video 8 In vivo two-photon imaging of Iba1-eGFP-labeled monocytes transiently adhering to pial vessels without obstructing blood flow in AD mice (with Texas Red in the blood) (see also Fig. 5d).

Supplementary Video 9 In vivo two-photon imaging of neutrophils stalling in a capillary branching from an AV in the barrel cortex of an AD mouse (see also Fig. 5f).

Supplementary Video 10 In vivo two-photon imaging of cerebral capillaries in AD mice treated for 1.5 months with nimodipine or vehicle (used to dissolve nimodipine) in the drinking water (see also Fig. 6c).

## Source data

Source Data Fig. 1 Statistical Source Data.

Source Data Fig. 2 Statistical Source Data.

Source Data Fig. 3 Statistical Source Data.

Source Data Fig. 4 Statistical Source Data.

Source Data Fig. 5 Statistical Source Data.

Source Data Fig. 6 Statistical Source Data.

Source Data Extended Data Fig. 1 Statistical Source Data.

Source Data Extended Data Fig. 2 Statistical Source Data.

Source Data Extended Data Fig. 4 Statistical Source Data.

Source Data Extended Data Fig. 5 Statistical Source Data.

Source Data Extended Data Fig. 6 Statistical Source Data.

Source Data Extended Data Fig. 7 Statistical Source Data.

Source Data Extended Data Fig. 8 Statistical Source Data.

Source Data Extended Data Fig. 9 Statistical Source Data.

Source Data Extended Data Fig. 10 Statistical Source Data.

## Data Availability

Data for each plot are available in Source Data files. Immunostaining images in Extended Data Fig. 3a–d and the lower panel in Extended Data Fig. 5a are available from ref. ^94^ (version 23, https://www.proteinatlas.org/). Left panels in Extended Data Fig. 3a–d and the upper panel in Extended Data Fig. 5a are from ref. ^43^ (https://betsholtzlab.org/VascularSingleCells/database.html). Data in Extended Data Fig. 3e are available from ref. ^95^ (https://twc-stanford.shinyapps.io/human_bbb/). Source data are provided with this paper.

## Code availability

To quantify microglial surveillance, we used code at https://github.com/AttwellLab/Microglia. To quantify vessel diameter, we used a FIJI macro provided by J. S. Y. Lim (A*STAR Microscopy Platform, Singapore), available at https://github.com/AttwellLab/Diameter.
