# Supplementary material for: Inhibiting Ca2+ channels in Alzheimer’s disease model mice relaxes pericytes, improves cerebral blood flow and reduces immune cell stalling and hypoxia
Source: Nat Neurosci. 2024 Sep 18;27(11):2086–100. doi: 10.1038/s41593-024-01753-w (PMC11537984; doi:10.1038/s41593-024-01753-w)
Supplement: Supplementary file 1 — Concentration of nimodipine used and mechanism of action. Supplementary References, Supplementary Fig. 1 and list of supplementary videos. [file 41593_2024_1753_MOESM1_ESM.pdf]

nature neuroscience

Supplementary information

https://doi.org/10.1038/s41593-024-01753-w

Inhibiting Ca^{2+} channels in Alzheimer's disease model mice relaxes pericytes, improves cerebral blood flow and reduces immune cell stalling and hypoxia

In the format provided by the authors and unedited

Concentration of nimodipine used and mechanism of action

We assume nimodipine acts by inhibiting voltage-gated calcium channels, but at high concentrations it has been suggested to also inhibit the production of NO and the inflammatory mediators TNFα, IL1β and COX2. The following considerations argue against these other pathways mediating the effects we report. We injected 220 μg/kg nimodipine (MW 418 g/mol) into mice of ~25 g weight (Extended Data Fig. 8b). Even if this were uniformly present in the extracellular space (~20% of the volume of the animal of density of 1/0.95 kg/L) its concentration would maximally be 220×10^{-6} g/kg x 0.025 kg/[418g/mol x (0.2 x 0.025kg x 0.95L/kg)] = 2.77 μM. This is 20 times the IC50 for blocking CaV1.2-based Ca^{2+} channels[97] (139 nM) and will block them by ~95%. Slow penetration across the BBB will probably result in a lower concentration. In contrast, the IC50 for nimodipine blocking NO production by microglia in response to LPS is ~12.8 μM (from fitting a curve (maximum response to LPS)/(1+[nimodipine]/IC50) to data in Fig. 5A of reference 97), and so 2.77 μM (or less) nimodipine would inhibit this by only 18% (or less). More importantly, this effect of nimodipine reflects it inhibiting iNOS expression[98], and so would presumably take hours to occur. This is inconsistent with nimodipine decreasing pericyte and SMC calcium level and increasing capillary and arteriole diameter within 3-10 minutes in Figs. 1h and 2d. In addition, the changes of vessel diameter with administration of nimodipine in the drinking water are similar to those seen when it is injected. Furthermore, NO and free radicals such as ONOO- derived from it are not involved[12] in the capillary constriction evoked by Aβ. We conclude that the acute effects of nimodipine on vessel diameter are not mediated by inhibition of iNOS expression. Similarly, the effects of nimodipine on production of the other inflammatory mediators TNFalpha, IL1β and COX2 all exhibit a similar high IC50 and reflect inhibition of synthesis of the relevant proteins[98]. Thus, it is implausible that the rapid effects of nimodipine on pericyte and SMC calcium level, and on capillary and arteriole diameter, could be mediated by these actions.

Supplementary References

97. Xu, W. & Lipscombe D. Neuronal Ca(V)1.3alpha(1) L-type channels activate at relatively hyperpolarized membrane potentials and are incompletely inhibited by dihydropyridines. *J. Neurosci.* **21**, 5944-5951 (2001).

98. Li, Y., Hu, X., Liu, Y., Bao, Y. & An, L. Nimodipine protects dopaminergic neurons against inflammation-mediated degeneration through inhibition of microglial activation. *Neuropharmacol.* **56**, 580-589 (2009).

Supplementary Fig. 1: Representative flow cytometry scatter plots highlighting the gating strategy for identifying neutrophils in whole blood.

a-d Gating on counting beads or whole cells, with the latter further refined into (**b**) single cells, (**c**) CD45^{+} leukocytes, and (**d**) CD45^{+}, Ly6G^{+} neutrophils.

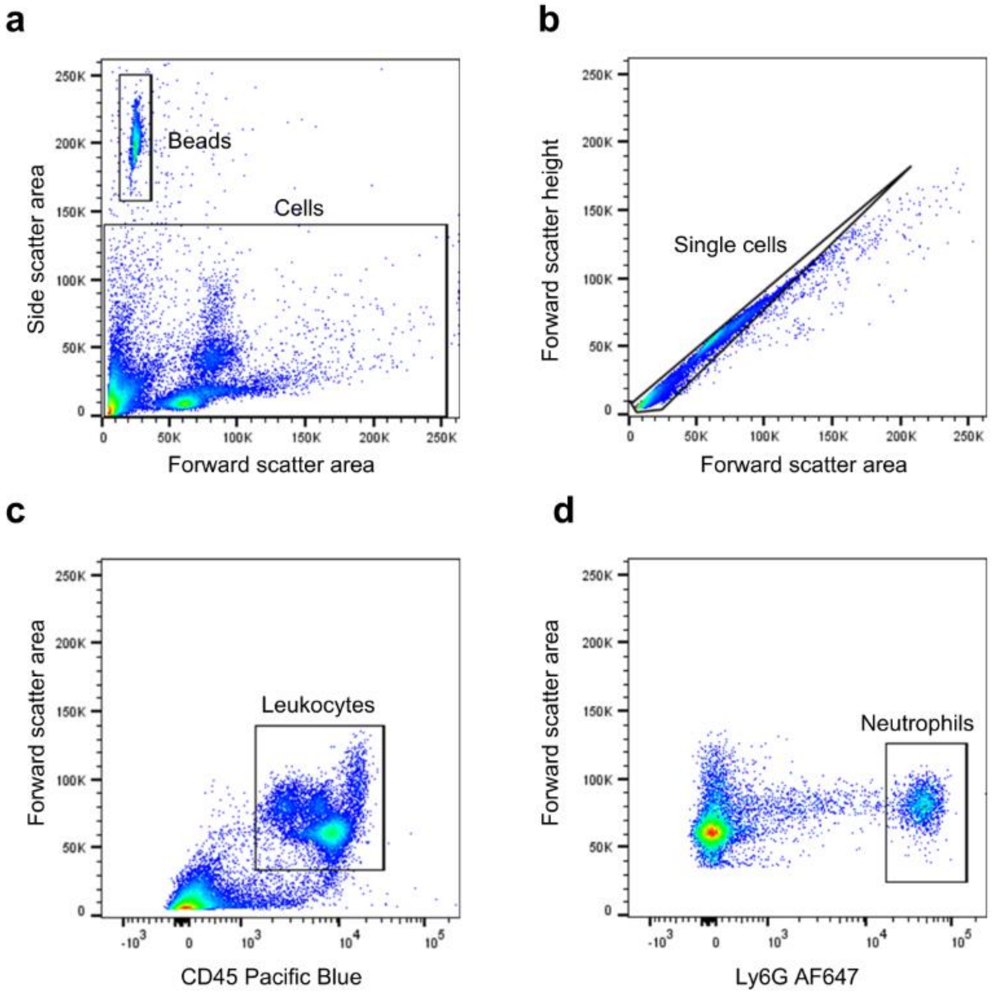

Supplementary Figure 1

Supplementary Videos

Supplementary Video 1

Pericytes of the 1st-3rd capillary branching order and >3rd capillary branching order with circumferential processes near their somata labeled using tdTomato (recoloured green) in cerebral cortex of wild-type NG2-Cre^{ERT2}-GCaMP5G mice in vivo, and rendered in IMARIS (see also Extended Data Fig. 1a).

Supplementary Video 2

Nimodipine reduces $[Ca^{2+}]_i$ in processes and somata of 1st branch order pericyte and dilates capillary in barrel cortex of Alzheimer's disease NG2-Cre^{ERT2}-GCaMP5G mouse (see also Fig. 2f).

Supplementary Video 3

In vivo two-photon imaging of pericyte with excitation at 940 nm or 800 nm in the barrel cortex of an Alzheimer's disease NG2-Cre^{ERT2}-GCaMP5G mouse (see also Extended Data Fig. 2e-g). Repeated Ca^{2+} transients seen in the image excited at 940 nm are absent in the image excited at 800 nm.

Supplementary Video 4

Laser-evoked injury raises $[Ca^{2+}]_i$ and contracts >3rd branch order pericytes near their somata in the barrel cortex of wild-type NG2-Cre^{ERT2}-GCaMP5G mice in vivo (see also Fig. 3d-h).

Supplementary Video 5

Laser-evoked injury does not raise $[Ca^{2+}]_i$ in penetrating arteriole smooth muscle cells and 2nd branch order capillary pericytes in barrel cortex of wild-type NG2-Cre^{ERT2}-GCaMP5G mouse in vivo (see also Extended Data Fig. 4b-d).

Supplementary Video 6

In vivo two-photon imaging of capillary blocks near pericytes labeled with NG2-dsRed in the barrel cortex of an AD mouse with FITC-dextran in the blood (see also Fig. 5a).

Supplementary Video 7

In vivo two-photon imaging of Ly6G-labeled neutrophils or Iba1-eGFP expressing monocytes in the vessel lumen (labeled with Texas Red in the blood) in barrel cortex of an AD mouse (see also Fig. 5d).

Supplementary Video 8

In vivo two-photon imaging of Iba1-eGFP labeled monocytes transiently adhering to pial vessels without obstructing blood flow in AD mice (with Texas Red in the blood) (see also Fig. 5d).

Supplementary Video 9

In vivo two-photon imaging of neutrophils stalling in a capillary branching from an ascending venule (AV) in the barrel cortex of an AD mouse (see also Fig. 5f).

Supplementary Video 10

In vivo two-photon imaging of cerebral capillaries in AD mice treated for 1.5 months with nimodipine or vehicle (used to dissolve nimodipine) in the drinking water (see also Fig. 6c).
