# Supplementary material for: Inhibiting Ca2+ channels in Alzheimer’s disease model mice relaxes pericytes, improves cerebral blood flow and reduces immune cell stalling and hypoxia
Source: Nat Neurosci. 2024 Sep 18;27(11):2086–100. doi: 10.1038/s41593-024-01753-w (PMC11537984; doi:10.1038/s41593-024-01753-w)
Supplement: Supplementary file 2 — Reporting Summary [file 41593_2024_1753_MOESM2_ESM.pdf]

Corresponding author(s): David Attwell, d.attwell@ucl.ac.uk; or Nils Korte, nils.korte@childrens.harvard.edu

Last updated by author(s): 29th June 2024

Reporting Summary

Nature Portfolio wishes to improve the reproducibility of the work that we publish. This form provides structure for consistency and transparency in reporting. For further information on Nature Portfolio policies, see our Editorial Policies and the Editorial Policy Checklist.

Statistics

For all statistical analyses, confirm that the following items are present in the figure legend, table legend, main text, or Methods section.

n/a	Confirmed	
☐	☒	The exact sample size (*n*) for each experimental group/condition, given as a discrete number and unit of measurement
☐	☒	A statement on whether measurements were taken from distinct samples or whether the same sample was measured repeatedly
☐	☒	The statistical test(s) used AND whether they are one- or two-sided<br>*Only common tests should be described solely by name; describe more complex techniques in the Methods section.*
☒	☐	A description of all covariates tested
☐	☒	A description of any assumptions or corrections, such as tests of normality and adjustment for multiple comparisons
☐	☒	A full description of the statistical parameters including central tendency (e.g. means) or other basic estimates (e.g. regression coefficient) AND variation (e.g. standard deviation) or associated estimates of uncertainty (e.g. confidence intervals)
☐	☒	For null hypothesis testing, the test statistic (e.g. *F*, *t*, *r*) with confidence intervals, effect sizes, degrees of freedom and *P* value noted<br>*Give P values as exact values whenever suitable.*
☒	☐	For Bayesian analysis, information on the choice of priors and Markov chain Monte Carlo settings
☒	☐	For hierarchical and complex designs, identification of the appropriate level for tests and full reporting of outcomes
☒	☐	Estimates of effect sizes (e.g. Cohen's *d*, Pearson's *r*), indicating how they were calculated

Our web collection on statistics for biologists contains articles on many of the points above.

Software and code

Policy information about availability of computer code

Data collection	Data were collected using ZEISS ZEN 2011, ZEISS ZEN 2.3 SP1 FP3 (black) and Olympus FV31S-SW - FluoView (for confocal and two-photon imaging), Matlab 2015b (for laser Doppler flowmetry), RWD Laser Speckle Imaging System software version 1.0, sCODA software version 4.1 (for blood pressure recordings), Diva 9.0.1 (for flow cytometry) and ParaVision 6.1 (for MRI).
Data analysis	Data analysis was performed using Matlab 2011b, 2018b and 2019b, FIJI (ImageJ 2.1.0), FlowJo 10 and IMARIS 9.1. Code used to analyse surveillance is available from https://github.com/AttwellLab/Microglia. To measure vessel diameters, we used FIJI. Statistical tests were performed in R (versions 3.4.2 or 3.6.3), Prism 6 or Prism 10 (GraphPad Software Inc., CA, USA).

For manuscripts utilizing custom algorithms or software that are central to the research but not yet described in published literature, software must be made available to editors and reviewers. We strongly encourage code deposition in a community repository (e.g. GitHub). See the Nature Portfolio guidelines for submitting code & software for further information.

Data

Policy information about availability of data

All manuscripts must include a data availability statement. This statement should provide the following information, where applicable:

- Accession codes, unique identifiers, or web links for publicly available datasets
- A description of any restrictions on data availability
- For clinical datasets or third party data, please ensure that the statement adheres to our policy

Source data are provided with this paper. Immunostaining images in Extended Data Fig. 3a-d and lower panel in Extended Data Fig. 5a are available from Ref. 95 (v23.proteinatlas.org). Left panels in Extended Data Fig. 3a-d and upper panel in Extended Data Fig. 5a is available from Ref. 43 (https://betsholtzlab.org/VascularSingleCells/database.html). Data in Extended Data Fig. 3e is available from Ref. 96 (https://twc-stanford.shinyapps.io/human_bbb/). All other data are available from the corresponding authors upon request.

Human research participants

Policy information about studies involving human research participants and Sex and Gender in Research.

Reporting on sex and gender	Sex- and gender-based analyses were not performed. Patients provided consent for information about their sex, age and diagnosis. Because of the low number of female (n=1) versus male patients (n=4) no statistical comparison between male and female patients was feasible.
Population characteristics	Patients were 17-65 years old and underwent neurosurgical glioma resection. No consent was obtained to collect data about past diagnoses, medications or genotypic information and therefore this information was not available.
Recruitment	Patients were recruited at the National Hospital for Neurology and Neurosurgery, Queen Square, London. Patients were recruited by the neurosurgeon attending to the patients. Patients of either sex and at least 16 years of age were selected for recruitment if they were scheduled to undergo neurosurgical glioma resection. Investigators performing the experiments were not involved in the patient recruitment process to remove biases in patient selection.
Ethics oversight	All work was performed with the informed consent of the patients and ethical approval from the National Health Service (REC number 15/NW/0568 and IRAS project ID 180727).

Note that full information on the approval of the study protocol must also be provided in the manuscript.

Field-specific reporting

Please select the one below that is the best fit for your research. If you are not sure, read the appropriate sections before making your selection.

☒ Life sciences ☐ Behavioural & social sciences ☐ Ecological, evolutionary & environmental sciences

For a reference copy of the document with all sections, see nature.com/documents/nr-reporting-summary-flat.pdf

Life sciences study design

All studies must disclose on these points even when the disclosure is negative.

Sample size	Sample sizes were chosen based on previous publications where possible. The increase in nimodipine-evoked CBF reported in anaesthetised monkeys (PMID: 7328148) was similar to the increase we report in WT mice. Previous work by our group using similar sample sizes reported on the decrease in capillary diameter at pericyte somata and the increase in hypoxic cells in the APP NL-G-F mice, as well as on the magnitude of the amyloid beta evoked constriction in live human tissue and the ET-1 evoked calcium rise in mouse tissue in the presence or absence of nimodipine or a TMEM16A blocker (PMID: 31221773, PMID: 35316222). Furthermore, the magnitude of the CBF decrease, percentage of cerebral capillary segments blocked by neutrophils and the calcium channel block evoked decrease in blood pressure were known for AD mouse models (PMIDs: 20479943, 23273599, 19398247, 25108425, 30742116, 14746921). No statistical test was performed to pre-determine sample size.
Data exclusions	Data with unstable cerebral blood flow (CBF) or significant changes in vessel diameter or pericyte [Ca2+]i prior to application of drugs were excluded. Mice with unsuccessful cardiac perfusions or poor cranial window preparations (resulting in unstable imaging) were excluded. Exclusion criteria were pre-established.
Replication	All representative experiments shown were repeated with similar results in at least 3 independent experiments. All attempts at replication were successful.
Randomization	Experiments using mice or live human tissue were randomly allocated to experimental groups.
Blinding	Investigators were blinded to group allocation during analysis and in some cases during data collection (e.g. MRI, immunohistochemistry) unless blinding was not feasible due to the autofluorescence of amyloid beta plaques present in the brains of AD mice.

Reporting for specific materials, systems and methods

We require information from authors about some types of materials, experimental systems and methods used in many studies. Here, indicate whether each material, system or method listed is relevant to your study. If you are not sure if a list item applies to your research, read the appropriate section before selecting a response.

Materials & experimental systems

n/a	Involved in the study
☐	☒ Antibodies
☒	☐ Eukaryotic cell lines
☒	☐ Palaeontology and archaeology
☐	☒ Animals and other organisms
☒	☐ Clinical data
☒	☐ Dual use research of concern

Methods

n/a	Involved in the study
☒	☐ ChIP-seq
☐	☒ Flow cytometry
☐	☒ MRI-based neuroimaging

Antibodies

Antibodies used	Primary antibodies: 82E1, IBL 10323 (clone: 820); P2Y12R, Anaspec AS-55043A; LAMP-1, BioLegend 121602 (clone: 1D4B); CD31,R&D AF3628; Fibrinogen, US Biological F4203-02F; CD206, R&D AF2535; ICAM-1, BioLegend 116114 (clone: YN1/1.7.4); VCAM-1, BioLegend 105712 (clone: 429 (MVCAM.A)); Ly6G, BioLegend 127610 (clone: 1A8); CD45, BioLegend 103125 (clone: 30-F11); ter119, BioLegend 116211 (clone: TER-119); gp91-phox AF647, Santa Cruz sc130543 (clone: 54.1), F4/80, BioLegend 123137 (clone: BM8). Secondary antibodies (from ThermoFisher): anti-mouse Alexa Fluor 405, A-31553; anti-mouse Alexa Fluor 488, A-21202; anti-mouse Alexa Fluor 633, A-21050; anti-rabbit Alexa Fluor 488, A21206; anti-sheep Alexa Fluor 633, A-21100; anti-goat Alexa Fluor 488, A-11055; anti-goat Alexa Fluor Plus 647, A32849; anti-rat Alexa Fluor 633, A-21094.
Validation	Validation data can be found on the manufacturer's website or in relevant citations, which were searched using the BenchSci platform (benchsci.com). Primary antibodies were validated as follows: 82E1, the manufacturer validated that 82E1 detects soluble and fibril Aβ and specifically reacts with the N-terminal of Aβ, but not with APP, Aβ(2-40) and Aβ(3-40) (https://www.ibl-america.com/amyloid-beta-n-82e1-a-anti-human-mouse-igg-moab-1/). 82E1 was also validated via IHC in brain tissue from APP NL-G-F mice (PMID: 24728269); P2Y12R, was validated via IHC in cortical tissue from P2Y12R knockout mice (PMID: 38622726); LAMP1 reactivity with recombinant LAMP-1 was confirmed by the manufacturer (https://www.biolegend.com/de-at/products/purified-anti-mouse-cd107a-lamp-1-antibody-3585) and the antibody was validated via IHC in brain tissue (PMID: 37024714); CD31 antibody was validated by IHC in brain tissue (PMID: 30498224); Fibrinogen antibody was validated by IHC in brain tissue (PMID: 35244976); CD206 antibody was validated by IHC in brain tissue (PMID: 36753421); ICAM-1 and VCAM-1 antibodies were validated in mouse endothelial cells (PMIDs: 38723627, 35510986); Ly6G antibody was validated for IHC by the manufacturer (https://www.biolegend.com/en-ie/products/alexa-fluor-647-anti-mouse-ly-6g-antibody-4780?GroupID=BLG5803); CD45 and ter119 antibodies were validated via flow cytometry (PMIDs: 33563975, 33667369); gp91-phox antibody was validated via IHC in mouse macrophages (PMID: 33841423); F4/80 antibody was verified for IHC by the manufacturer (https://www.biolegend.com/de-at/products/brilliant-violet-421-anti-mouse-f4-80-antibody-7199).

Animals and other research organisms

Policy information about studies involving animals; ARRIVE guidelines recommended for reporting animal research, and Sex and Gender in Research

Laboratory animals	Mice were bred on a C57/BL6J (Charles River) background and aged P22-P480. Mice (either sex) in individually-ventilated cages (12-hour light/dark cycle, 21±1°C, 45-65% humidity) were fed normal chow and tap water ad libitum. Cages contained Aspen chip and Sizzle-Nest bedding and an environmental enrichment tunnel. APP NL-G-F NG2-dsRed mice were obtained by crossing NG2-dsRed mice (obtained by their generator Akiko Nishiyama (University of Connecticut, USA) via Dirk Dietrich (University Clinic Bonn, Germany); they can be obtained from Jackson Laboratories, #008241) with APP NL-G-F mice (obtained from their generator Takaomi Saido, RIKEN, Japan). AD x Iba1-eGFP mice or AD x CX3CR1-GFP mice were obtained by crossing APP NL-G-F mice with Iba1-eGFP mice (obtained from their generator Shinichi Kohsaka, National Institute of Neuroscience, Japan) or heterozygous CX3CR1-GFP mice (provided by Soyon Hong, University College London, UK); they can be obtained from Jackson Laboratories, #005582), respectively. AD x NG2-CreERT2-GCaMP5G mice were generated by crossing AD mice with tamoxifen-inducible NG2-CreERT2 knock in mice (provided by Frank Kirchhoff, University of Saarland, Germany) and floxed GCaMP5G-IRESTdTomato mice (Jackson Laboratories, #024477).
Wild animals	No wild animals were used in this study.
Reporting on sex	The sex of mice was recorded for all mice. Sex-based analyses were not performed as it was not a primary focus of the current study.
Field-collected samples	No field samples were used in this study.
Ethics oversight	Animal breeding, experimental procedures and killing methods were in accordance with UK Home Office regulations (Guidance on

Ethics oversight	the Operation of Animals, Scientific Procedures Act, 1986) and the advice of the UCL Animal Welfare Ethical Review Board, or overseen by Boston Children's Hospital Institutional Animal Care and Use Committee following NIH guidelines.

Note that full information on the approval of the study protocol must also be provided in the manuscript.

Flow Cytometry

Plots

Confirm that:

☒ The axis labels state the marker and fluorochrome used (e.g. CD4-FITC).

☒ The axis scales are clearly visible. Include numbers along axes only for bottom left plot of group (a 'group' is an analysis of identical markers).

☒ All plots are contour plots with outliers or pseudocolor plots.

☒ A numerical value for number of cells or percentage (with statistics) is provided.

Methodology

Sample preparation	The tail vein of mice was nicked using a 29G needle, and whole blood collected into PBS containing 5 mM EDTA. Following removal of supernatant after centrifiguation at 400 g for 5 minutes at 4°C, cells were incubated with ACK lysis buffer (150 mM NH4Cl, 1 mM KHCO3, 0.1 mM EDTA) for 5 minutes at RT under gentle agitation and MACS buffer was added (2% serum, 1 mM EDTA, PBS). Supernatant was removed after centrifugation at 400 g for 5 minutes and cells were incubated with Pacific Blue anti-mouse CD45 (2.5 µg/ml, Biolegend 103125) or Alexa Fluor 647 anti-mouse Ly6G (2.5 µg/ml, BioLegend 127610) antibodies for 30 minutes at 4°C in the dark. Supernatant was removed after centrifugation at 400 g for 5 minutes. Cells were washed twice in MACS buffer by removing supernatant (as before) and 123count eBeads counting beads (Thermo Fisher Scientific, # 01-1234-42) were added to the sample to count the live cells using a BD LSRFortessa™ Cell Analyzer.
Instrument	BD LSRFortessa™ Cell Analyzer
Software	Diva 9.0.1 was used to collect data and Flowjo version 10 was used to analyse data.
Cell population abundance	Cell sorts were not conducted as part of this work and therefore cell purity is not applicable. For detail, neutrophils constituted approximately 15% of the total leukocyte pool in the whole blood of mice as analysed using the shown gating strategy using FlowJo software.
Gating strategy	The cellular fraction of whole blood was initially identified by the forward and side scatter profiles. Single cells were then identified by the forward scatter height, and forward scatter area profiles. All leukocytes were identified by the use of a CD45 Pacific blue fluorescently conjugated antibody and neutrophils were further identified by double positivity of CD45 (pacific blue), and Ly6G (AF647), the latter marker exclusively identifying neutrophils. Counting beads were identified by their high side scatter profile. The boundaries used for such gating strategies were clear, as can be seen in the accompanying figure.

☒ Tick this box to confirm that a figure exemplifying the gating strategy is provided in the Supplementary Information.

Magnetic resonance imaging

Experimental design

Design type	Multi-TI ASL FAIR scan to estimate CBF within a single coronal slice of the anaesthetised mouse brain
Design specifications	NA
Behavioral performance measures	NA

Acquisition

Imaging type(s)	Multi-TI ASL FAIR scan to estimate CBF within a single coronal slice of the anaesthetised mouse brain
Field strength	9.4T
Sequence & imaging parameters	Multi-TI ASL FAIR, single slice spin-echo EPI readout (single shot).
Area of acquisition	Single coronal slice chosen based on visial inspection and manual positioning of an anatomical reference images (T2-weighted FSEMS).

Diffusion MRI ☐ Used ☒ Not used

Preprocessing

Preprocessing software	NA
Normalization	NA
Normalization template	NA
Noise and artifact removal	NA
Volume censoring	NA

Statistical modeling & inference

Model type and settings	The ASL and control signal were taken within ROIs that were manually drawn. The control signal was fitted to a standard inversion recovery model to estimate T1 and M0. The ASL signal was fitted to a single compartment Buxton model to extract CBF.
Effect(s) tested	NA

Specify type of analysis: ☐ Whole brain ☒ ROI-based ☐ Both

Anatomical location(s): ROIs were manually drawn on the raw images.

Statistic type for inference (See Eklund et al. 2016)	*Specify voxel-wise or cluster-wise and report all relevant parameters for cluster-wise methods.*
Correction	*Describe the type of correction and how it is obtained for multiple comparisons (e.g. FWE, FDR, permutation or Monte Carlo).*

Models & analysis

n/a	Involved in the study
☒	☐ Functional and/or effective connectivity
☒	☐ Graph analysis
☒	☐ Multivariate modeling or predictive analysis
